# Apurinic/apyrimidinic endodeoxyribonuclease 1 (APE1) promotes stress granule formation via YBX1 phosphorylation in ovarian cancer

**DOI:** 10.1007/s00018-023-05086-y

**Published:** 2024-03-04

**Authors:** Shuyu Mao, Chong Xie, Yufeng Liu, Ye Zhao, Mengxia Li, Han Gao, Yue Xiao, Yongkang Zou, Zhiguo Zheng, Ya Gao, Juan Xie, Bing Tian, Liangyan Wang, Yuejin Hua, Hong Xu

**Affiliations:** 1 MOE Key Laboratory of Biosystems Homeostasis and Protection, Institute of Biophysics, College of Life Science, Zhejiang University, Hangzhou, China; 5 Institute of Pathology, University Medical Center Göttingen, Göttingen, Germany; 2 Institute for Cancer Research, Shenzhen Bay Laboratory, Shenzhen, 518107 China; 3 Department of Cancer Center, Daping Hospital, Army Medical University, Chongqing, China; 4 The Cancer Hospital of the University of Chinese Academy of Sciences (Zhejiang Cancer Hospital), Institute of Basic Medicine and Cancer (IBMC), Chinses Academy of Sciences, Hangzhou, China

**Keywords:** APE1, Stress granule, Phase separation, YBX1, Phosphorylation

## Abstract

APE1 is an essential gene involved in DNA damage repair, the redox regulation of transcriptional factors (TFs) and RNA processing. APE1 overexpression is common in cancers and correlates with poor patient survival. Stress granules (SGs) are phase-separated cytoplasmic assemblies that cells form in response to environmental stresses. Precise regulation of SGs is pivotal to cell survival, whereas their dysregulation is increasingly linked to diseases. Whether APE1 engages in modulating SG dynamics is worthy of investigation. In this study, we demonstrate that APE1 colocalizes with SGs and promotes their formation. Through phosphoproteome profiling, we discover that APE1 significantly alters the phosphorylation landscape of ovarian cancer cells, particularly the phosphoprofile of SG proteins. Notably, APE1 promotes the phosphorylation of Y-Box binding protein 1 (YBX1) at S174 and S176, leading to enhanced SG formation and cell survival. Moreover, expression of the phosphomutant YBX1 S174/176E mimicking hyperphosphorylation in APE1-knockdown cells recovered the impaired SG formation. These findings shed light on the functional importance of APE1 in SG regulation and highlight the importance of YBX1 phosphorylation in SG dynamics.

**Supplementary Information:**

The online version contains supplementary material available at 10.1007/s00018-023-05086-y.

## Introduction

Cancer cells are constantly under threat from both intercellular and extracellular stress ranging from endogenous reactive oxygen species (ROS) to therapeutic drugs. Cells developed an intricate network of defense mechanisms to combat adverse conditions. These adaptations become even more elaborate and diversified in cancer cells to guarantee cell survival under the hostile tumor microenvironment and the lethal chemicals. Ovarian cancer (OC) is a highly fatal disease and ranks as the second leading cause of death among gynecological cancers [1]. Anti-cancer drugs such as platinum analogues, the most active therapeutic agents against OC, induce a massive production of ROS that leads to oxidative stress (OS), causing oxidative DNA damage and apoptosis [2]. To cope with OS, cancer cells adapt through mechanisms such as SG formation. To this day, platinum resistance remains the biggest challenge in OC treatment [3], whereas SGs are found to facilitate chemoresistance acquisition [4, 5].

SGs are a class of ribonucleoprotein (RNP) granules that form non-membrane bound cellular compartments through phase separation [6]. The formation of SGs is an essential stress-coping mechanism that protects cells from both endogenous and exogeneous stress, including anti-cancer drugs. Oxidative stress is a common inducer of SGs, where ROS, such as H_2_O_2_, is routinely used to induce SG formation [7]. The primary function of SGs is to inhibit general translation, suppressing most housekeeping genes to allow efficient and selective production of stress response factors [8]. Therefore, the precise regulation of SGs is vital for cell survival [9]. Phosphorylation is the most extensively studied posttranslational modifications (PTMs) that actively modulates SG dynamics [10–12]. For example, phosphorylation of eukaryotic initiation factor 2α (eIF2α) at Ser51 (S51) is crucial for SG formation under multiple types of stress [13, 14]. Conversely, phosphorylation of tristetraprolin (TTP) prevents its recruitment to SGs and regulates the interaction between SGs and processing bodies (PBs) [15]. Moreover, the phosphorylation of growth factor receptor-bound protein 7 (Grb7) accelerates the disassembly of heat shock-induced SGs [16], while phosphorylation of the SG-nucleating protein SH3-domain-binding protein 1 (G3BP1) at serine 149 (S149) impairs its ability for SG induction [17, 18]. In addition, YBX1, a strong RNA-binding protein (RBP), is also found to participate in SG regulation. YBX1 binds to and translationally activates the 5′ untranslated region (UTR) of G3BP1 mRNAs to promote SG assembly [19]. What’s more, YBX1 is observed in SGs in U2OS cells [19] and zebrafish cells [20]. Whether phosphorylation of YBX1 is engaged in SG regulation is unknown.

Further, YBX1 has been shown to interact with acetylated APE1 to enhance the expression of multidrug resistance 1 (MDR1) [21] to facilitate drug resistance. APE1, a multi-functional protein, is frequently overexpressed in OC and other cancer types [22, 23]. APE1 overexpression is associated with poor patient survival as it contributes to cancer progression by promoting cancer cell proliferation, migration, and chemoresistance [10, 24–30]. Functionally, APE1 participates in base excision repair (BER) and nucleotide excision repair (NER) to repair damaged DNA [31]. Moreover, APE1 activates TFs such as activator protein 1 (AP-1), nuclear factor kappa B (NF-κB), hypoxia-inducible factor 1 (HIF-1), and cAMP response element-binding protein 1 (Creb1) to facilitate cell proliferation and cell survival under stress [32–34]. Recent findings also reported roles of APE1 in RNA metabolism such as miRNA processing [35]. However, whether APE1 exerts other regulatory roles to foster chemoresistance is unknown. Interestingly, several studies have suggested the involvement of APE1 in PI3K/AKT/mTOR signaling pathway [36], ERK1/2 signaling pathway [37] and MAPK signaling pathway [38, 39]. Despite our unpublished work and other studies demonstrating a handful of proteins or protein kinases whose phosphorylation is under the regulation of APE1, no research has evaluated how APE1 influences the cellular phophoproteome and more importantly, interrogated whether APE1 engages in stress response via modulating SGs. To investigate whether and how APE1 enhances cell survival through phosphorylation would expand our understanding of the functional role of APE1.

In this study, we discover that APE1 significantly alters the phospho-landscape of ovarian cancer cells, particularly the phosphoprofile of SG proteins to enhance SG formation and the redox function of APE1 is mainly responsible for SG regulation. Mechanistically, APE1 may facilitate dual phosphorylation of YBX1 at S174 and S176 via modulation of phosphatase PPP1R12A and kinase PLK1 to enhance SG assembly and cell survival. Our research reveals the active presence of APE1 in SGs and underscores the significant role of APE1 in SG regulation. These findings open the possibility for targeting APE1 as a therapeutic strategy for cancer and other SG-related diseases.

## Methods and materials

### Patients and tissue samples

A total of 25 patients were included in this study who were operated on for OC between 2018 and 2023 in the Cancer Center of Daping Hospital. Each ovarian tumor was histologically confirmed by a pathologist of Daping Hospital. Patients undergoing surgery were consecutively included in the patient cohort. According to the progression-free survival (PFS), OC patients who received cisplatin chemotherapy were divided into the cisplatin sensitive group (n = 13, PFS > 12 months) and the cisplatin resistant group (n = 12, PFS ≤ 6 months). The study was performed following the regulations of the ethics committee of Daping Hospital.

### Immunohistochemistry and scoring

Sections from paraffin-embedded tissues were deparaffinized by xylene and rehydrated through an ethanol series. For antigen retrieval, the slides were autoclaved in 1 mM EDTA buffer. Sections were then pre-incubated in PBS twice and incubated overnight at 4 °C with anti-APE1 antibody (Abcam). The sections were rinsed with PBS twice and incubated with its associated horseradish peroxidase (HRP)-conjugated secondary antibody for 30 min at room temperature (RT). Sections were rinsed with PBS and developed with diaminobenzidine (DAB) substrate, and counterstained with hematoxylin for nuclear staining. Positive staining was detected as a brown color.

A "quickscore" method for immunohistochemical semi-quantification was used to evaluate the expression level of APE1 [40]. Briefly, the proportion of cells with positive staining was termed category A where A was assigned scores from 1 to 6 (A = 1 (0–4%); 2 (5–19%); 3 (20–39%); 4 (40–59%); 5 (60–79%); 6 (80–100%)). Category B represents intensity of the staining, where B ranges from 0 to 3 (0 (negative); 1 (weak); 2 (moderate); 3 (strong)). The final score was calculated by multiplying A by B (ranging from 0 to 18). The average score of 4 images for each case was obtained for statistical analysis.

### Plasmids construction and site-directed mutagenesis

cDNA of *APE1* and *YBX1* were amplified via reverse transcription by polymerase chain reaction (PCR) from total mRNA extracted from 293 T cells. The Kozak sequence or 3×FLAG-tag is added to the N-terminal of *APE1.* The 3×FLAG-tag is added to the N-terminal of *YBX1*. Kozak-APE1 was cloned into a pCDH-CMV lentiviral vector while 3×Flag-APE1 or 3×Flag-YBX1 was constructed into a pcDNA 3.1 vector. For the knockdown of APE1, the sgRNA was designed using CHOPCHOP (http://chopchop.cbu.uib.no/). DNA oligos were synthesized and ligated into the lentiCRISPR v2 plasmid (Addgene). Mutations of YBX1 at S174 and S176 and mutations of APE1 at C65S and E96A were generated using the QuickChange II XL site-directed mutagenesis kit following the protocol of the manufacturer (Agilent Technologies). For in vitro phase separation assay, 6×His-mEGFP-YBX1 was constructed into a pET-28a plasmid. For live cell imaging, mEGFP-YBX1 was constructed in MigR1 plasmid. All sequences and mutations were confirmed by sequencing.

### Cell culture and construction of stable cell lines

SKOV3 and A2780 cells were cultivated in DMEM medium (Gibco) supplemented with 100 μg/ml penicillin, 100 μg/ml streptomycin (Gibco), and 10% fetal calf serum (FBS, Excel Bio). 293 T cells were cultivated in RPMI 1640 medium (Gibco), supplemented with 100 μg/ml penicillin, 100 μg/ml streptomycin, and 10% FBS. Cell lines were maintained at 37 °C in a humidified incubator with 5% CO_2_. To generate stable cell lines, the lentiviral plasmid pCDH-CMV containing Kozak-APE1 and lenti-CRISPR v2 plasmid containing sgRNA along with other packaging plasmids were transfected into 293 T cells to produce viruses. Viruses were collected 48–72 h post transfection and concentrated using Lenti-X Concentrator (Takara). SKOV3 and A2780 cells were infected with viruses and further selected with 1 μg/ml or 2 μg/ml of puromycin, as the lentiviral vector constructs harbor the puromycin resistance gene. Protein expression is confirmed by MS and WB analysis.

### Proliferation assay

For the proliferation assay, 1000 cells were seeded in a 96-well plate and cultured for 0, 24 h, 48 h and 72 h. Cell viability was assessed by adding 10 μl of cell-counting solution (CCK-8, Beyotime) to each well and incubating for 2 h. The absorbance was measured at 490 nm with a microplate reader (BioRad).

### Transwell assay

For the transwell assay, 5000 cells in 400 μl of serum-free medium were seeded into the transwell chambers with 8-μm pores (Corning) in a 24-well plate. The lower chambers were filled with 0.5 ml of DMEM supplemented with 10% FBS. Cells were cultured for 24 h before fixation with 4% paraformaldehyde (Beyotime) and stained with crystal violet solution (Beyotime). Images were taken using an inverted light microscopy (Leica).

### Cell viability assay

To assess cell viability after drug treatment, cells were seeded in 96-well plates at 5000 cells/well overnight and treated with varying concentrations of MMS (Sigma Aldrich), cisplatin (Beyotime) or H_2_O_2_ (Sigma Aldrich) for 18 h, 24 h, and 12 h respectively. Cell viability was determined using CCK-8 (Beyoime) following the manufacturer’s instructions. The absorbance was measured by a microplate reader (BioRad) at 490-nm wavelength. Cell viability was calculated as the percentage of staining intensity in treated groups relative to control. Four replicates were averaged for each treatment.

### Subcellular fractionation

Subcellular fractionation was conducted following the protocol by Viacheslav et al. with minor modifications [41]. Briefly, cells were harvested by trypsinization and collected by centrifugation at 1500 rpm at 4 °C for 4 min. Cell pellet was washed with ice-cold PBS and resuspended in ice-cold hypotonic buffer (20 mM Tris–HCl, pH 7.4, 10 mM KCl, 2 mM MgCl_2_, 1 mM EGTA, 0.5 mM DTT, 0.5 mM PMSF) and incubate on ice for 3 min. NP-40 was added (0.1%) to lyse cells. The sample was incubated on ice for 3 min and centrifuged at 1000 rpm at 4 °C for 5 min to separate the nuclei and cytoplasm. Nuclei were resuspended in an isotonic buffer (20 mM Tris–HCl, pH 7.4, 150 mM KCl, 2 mM MgCl_2_, 1 mM EGTA, 0.5 mM DTT, 0.5 mM PMSF, 0.2% NP-40) and incubate on ice for 10 min followed by centrifugation at 1000 rpm at 4 °C for 3 min (nuclear fraction). To collect the cytoplasmic fraction, the supernatant was centrifuged at 15,000 rpm at 4 °C for 3 min to pellet debris. The supernatant was collected (cytoplasmic fraction). The purity of each fraction was assessed by WB against the cytoplasmic marker GAPDH and nuclear marker Histone H3.

### Western blotting

To analyze protein by WB, cells were lysed in lysis buffer (Beyotime) supplemented with protease inhibitor (Promega) on ice for 30 min, followed by brief sonication. After clarification by centrifugation, protein quantification of the supernatant was measured by BCA assay (Beyotime). Equal amounts of total protein were loaded onto the SDS-PAGE gel for protein separation and transferred to a 22 μm PVDF membrane (Beyotime). The membrane was blocked with 5% nonfat milk for 1 h at RT and blotted with primary antibodies overnight at 4 °C. After washing the membrane 5 times for 5 min, the membrane was incubated with HRP-conjugated secondary antibodies. Chemiluminescence was detected with the ECL reagents (Solarbio) on X-ray films. The following primary antibodies were used: anti-APE1 (1:1000; Santa Cruz Biotechnology), anti-Actin (1:5000; Proteintech), anti-Flag-tag (1:1000; GeneScript), anti-YBX1 (1:1000; Cell Signaling Technology), anti-phosphoserine (1:1000; Abcam), anti-phospho-eIF2α (1:1000; Cell Signaling Technology), anti-eIF2α (1:1000; Cell Signaling Technology), anti-G3BP1 (1:2000; Proteintech), anti-PLK1 (1:1000, Proteintech) and anti-PPP1R12A (1:1000, Proteintech) antibodies.

### SG induction and immunofluorescence analysis

Cells were grown on coverslips overnight and treated with 0.5 mM H_2_O_2_ (Sigma-Aldrich) for 3 h or 250 μM cisplatin (Beyotime) for 4 h to induce SGs. After rinsing with PBS, cells were fixed in 4% paraformaldehyde for 8 min at RT, rinsed by PBS for three times, followed by incubation in 0.2% Triton X-100 for 8 min at RT and rinsed for three times with PBS. The coverslips were then blocked by 2% BSA for 1 h at RT and incubated with primary antibodies at 4 °C overnight. Primary antibodies used: anti-G3BP1 (1:200; Proteintech), anti-p-eIF2α (1:100; Cell Signaling Technology), anti-YBX1 (1:100; Cell Signaling Technology), anti-APE1 (1:100; Santa Cruz Biotechnology) and anti-Flag-tag (1:100; GeneScript). After rinsing with PBS, coverslips were incubated with Alexa Fluor 488-conjuaged secondary antibody (1:100; Thermo Fisher) or Alexa Fluor 674-conjugated secondary antibody (1:100; YiSheng) at RT for 1 h. Coverslips were then rinsed with PBS for three times and rinsed with ddH_2_O, dried and mounted in Prolong Glass Antifade Mountant (with Hoechst 33342; Invitrogen). Images were captured using a Nikon fluorescent microscope and analyzed by Image J software. Cells containing more than 2 SG foci were counted as SG-containing cells [42].

### Co-immunoprecipitation assay

Cells cultured in 10-cm plates of 95% confluency were lysed in co-immunoprecipitation lysis buffer (Beyotime), briefly sonicated and centrifuged for clarification. Proteins were quantified using a BCA assay kit, then incubated with either agarose beads (Santa Cruz Biotechnology) and the intended antibodies including anti-APE1 antibody (Santa Cruz Biotechnology), anti-YBX1 antibody (Cell Signaling Technology), anti-PLK1 antibody (Proteintech), and anti-PPP1R12A antibody (Proteintech) or IgG (Proteintech) or with Anti-flag M2 beads (Sigma-Aldrich) at 4 °C overnight. Beads were washed four times with co-immunoprecipitation buffer with rotation at 4 °C for 5 min each time, boiled with 5× SDS loading buffer (Beyotime), clarified by centrifugation and loaded onto an SDS-PAGE gel for WB analysis as described above. For detecting interacting proteins in the cytoplasm, the cytoplasmic fraction obtained by subcellular fractionation was used.

### Mass spectrometry analysis

Sample preparation for phosphoproteomic analysis was performed according to the EasyPhos protocol [43]. Briefly, cells were seeded in 6-well plates, lysed with sodium deoxycholate (SDC; Sigma-Aldrich; SDC buffer containing 4% (wt/vol) SDC and 100 mM Tris–HCl, pH 8.5), denatured by heating at 95 °C for 5 min, and then briefly sonicated. After clarification by centrifugation, protein content was quantified by BCA assay. 500 μg of protein was used for each sample. Reduction and alkylation were done using 100 mM Tris (2-carboxyethyl) phosphine hydrochloride (TCEP-HCl; Pierce) and 400 mM 2-Chloroacetamide (CAM; Sigma-Aldrich) at 45 °C for 5 min, followed by enzymatic digestion with trypsin (Sigma-Aldrich) at 37 °C for 18 h. Phosphopeptides were enriched with TiO_2_ beads (GL Sciences), followed by desalting with C18 stage tips (Pierce). For proteomic analysis, 500 μg of protein in SDC buffer was reduced and alkylated as beforementioned. Tryptic digestion was performed with trypsin (Sigma-Aldrich) at 37 °C for 18 h. Acid precipitation was conducted with 2% (v/v) trifluoroacetic acid (TFA) to remove SDC [44]. After centrifugation, pellet was washed for three times using 0.5% TFA with sonication. Peptides were desalted with C18 stage tips (Pierce). Samples were dried in an evaporative concentrator (Thermo Fischer) and reconstituted in MS loading buffer consisting of 0.3% (vol/vol) TFA/2% (vol/vol) ACN.

The samples were analyzed with an Orbitrap Fusion Lumos Tribrid mass spectrometer (Thermo Scientific) coupled with a nano-ESI source with the vendor-provided Tune and Xcalibur 4.3 software. Peptides were separated on a commercial RP-HPLC pre-column (75 μm × 2 cm) (Thermo, #164946) followed by a commercial RP-HPLC analytical column (75 μm × 25 cm) (Thermo, #164941), both packed with 2 μm C18 beads and connected to an EASY-nLC 1200 UHPLC system (Thermo). Peptides were separated by a 90-min LC gradient from 3 to 41% buffer B (80% (vol/vol) acetonitrile (ACN, Sigma Aldrich)/0.1% (vol/vol) formic acid (FA, Sigma Aldrich)), followed by a washout of 72% (vol/vol) ACN for 10 min [43]. Data were acquired by the Orbitrap Fusion Lumos via data-dependent acquisition (DDA). The spray voltage was set at 2.1 kV, while the temperature of the ion transfer capillary was 320 °C. The MS spectra from 350 to 2000 m/z were collected with 120,000 resolution, AGC of 4 × 10^5^ and maximal injection time at 150 ms. Top ten most abundant precursors (multiply charged) from each full scan went through fragmentation by higher energy collision dissociation (HCD) with 30% normalized collision energy. Dynamic exclusion was set to 30 s. Proteome Discoverer 2.5 software was used to perform label-free quantitative (LFQ) phosphoproteomic and proteomic analysis. The mass spectrometry proteomic data have been deposited to the ProteomeXchange Consortium via the PRIDE partner repository with the dataset identifier PXD040041.

### Protein expression and purification

Protein expression and purification were done using *Escherichia coli* strain BL21 (DE3; Transgene). Transformed *E. coli* BL21 clones were grown at 37 °C in LB medium with 50 μg/ml Kanamycin (Beyotime) to OD_600_ of 0.6–0.8. Isopropyl-β-d-thiogalactopyranoside (IPTG; Sigma Aldrich) was added to a final concentration of 0.4 mM to induce protein expression at 16 °C for 16 h. Bacteria were lysed in lysis buffer (20 mM Tris pH 7.8, 500 mM NaCl, 5% glycerol (Sigma Aldrich), 3 mM β-ME (Sigma Aldrich), and 10 mM imidazole (Sigma Aldrich)) with sonication, and centrifuged at 18,000×*g* for 45 min at 4 °C. The supernatant was purified using Ni-IDA beads resin (ProbeGene). Briefly, the resin was equilibrated with buffer A (20 mM Tris, pH 7.8, 500 mM NaCl, 5% w/v glycerol, and 10 mM imidazole). The supernatant was incubated with Ni-IDA beads at 4 °C for 30 min with rotation. After centrifugation, the resin was the washed with buffer A for three times, and eluted with buffer B (buffer A with 300 mM imidazole). Protein was desalted and concentrated with a 30 K molecular weight cutoff (MWCO) protein concentrator (Pierce), quantified and aliquoted.

### In vitro phase separation assay

Freshly purified proteins were diluted to a final concentration of 5 μM in phase separation buffer (25 mM Tris–HCl, pH 7.4, 150 mM KCl, 5% polyethylene glycol (PEG; Sigma Aldrich)) and incubated at RT for 30 min [45]. Zeiss LSM900 confocal microscope was used for imaging.

### Fluorescence recovery after photobleaching (FRAP) assay

FRAP was performed on 293 T cells transfected with mEGFP-YBX1 on Zeiss LSM900 confocal microscope. A circular region of ~ 1 µm in diameter was chosen and bleached for 5 iterations at 488 nm and stopped on intensity below 20%. A single pre-bleaching image (T0) was taken. After bleaching, images were taken every 3 s over 5 min. The FRAP curves were calculated by normalizing fluorescence signal to the background and the fluorescence intensity at T0 [46]. A minimum of three independent FRAP experiments were performed. Data was analyzed with FrapBlot (http://frapbot.kohze.com/) [47].

### In vitro phosphorylation and phase separation assay

Cells were lysed with lysis buffer (50 mM Tris–HCl, pH 7.0, 0.5% NP-40, protease inhibitor cocktail (Promega) and 2.5% RNase inhibitor (Transgene)) and incubated on ice for 10 min followed by brief sonication [48]. Cell lysate was clarified by centrigation at 15,000 rpm for 5 min at 4 °C. Phosphorylation reaction buffer contains 10 mM MgCl_2_, 50 μM ATP, 1 mM DTT and 50% cell lysate. 20 μM of mEGFP-YBX1 and 30 μM of PLK1 were added to the reaction buffer and incubated at 37 °C for 30 min to allow PLK1 to phosphorylate YBX1. The phase saperation assay was performed by adding 20 μM of mCherry-APE1 and 10 μM of mEGFP-YBX1 from the phosphorylation reaction to cell lysate. The reaction was kept at RT for 30 min before imaging.

### Statistical analysis

Statistical analysis was performed using GraphPad Prism. The data are presented as means ± SEM. Mean values were statistically analyzed by Student’s t test, one-way ANOVA, or two-way ANOVA. Multi-comparison was done using Dunnett's test. Statistical significance is denoted as *p < 0.05, **p < 0.01, or ***p < 0.001. All experiments were repeated for at least three times.

## Results

### APE1 promotes SG formation and cancer cell survival in ovarian cancer

**Fig. 1:**
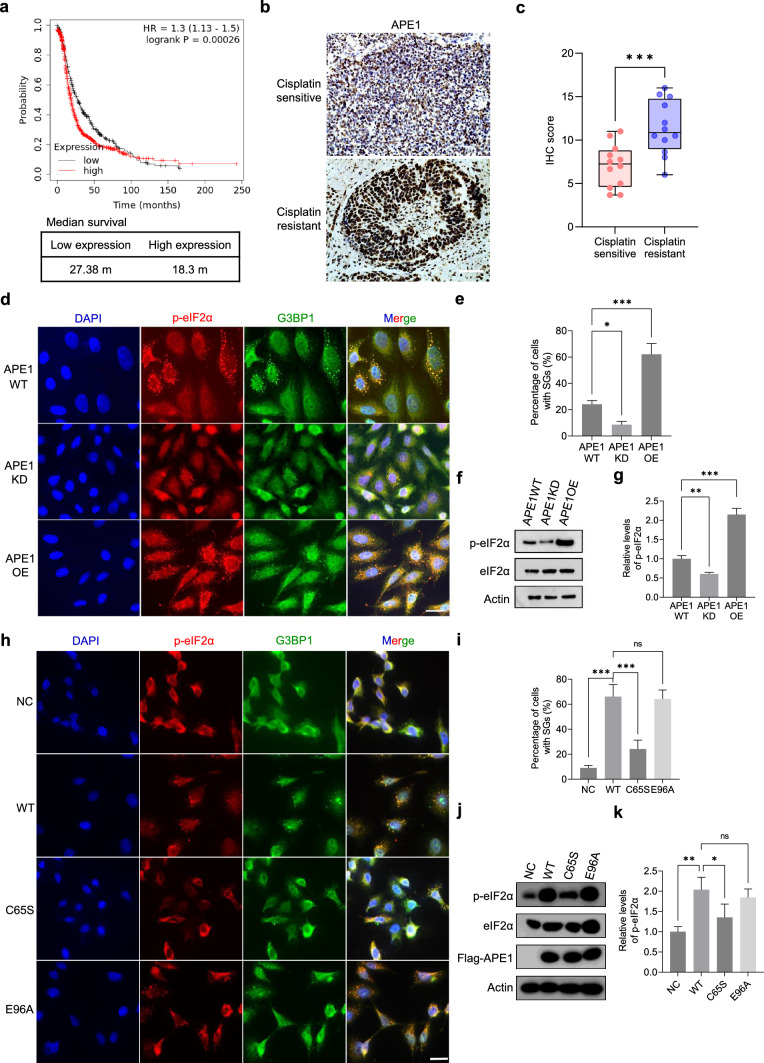
APE1 promotes SG formation and cell survival under stress. **a** Kaplan–Meier curve showing progression-free survival (PFS) of 1435 ovarian cancer patients with high or low APE1 mRNA level. Kaplan–Meier curve was created with Kaplan–Meier Plotter (https://kmplot.com/analysis/). **b** Representative IHC staining of APE1 expression in cisplatin-sensitive and cisplatin-resistant tumor tissues collected from ovarian cancer patients. Scale bar = 200 μm. **c** Immunohistochemistry score of APE1 expression in the cisplatin-sensitive and cisplatin-resistant groups. **d** Representative images of immunofluorescence (IF) studies conducted in APE1WT, APE1KD and APE1OE cells after 0.5 mM H_2_O_2_ treatment for 3 h. Coverslips were probed with p-eIF2α and G3BP1 antibodies to mark SGs. p-eIF2α (red) and G3BP1 (green) were merged with the nuclear stain DAPI (blue). Scale bar, 20 μm. **e** Quantification of the percentage of cells containing SGs in APE1KD and APE1OE relative to APE1WT. Data represent means ± SEM of n = 3 independent replicates. **f** Representative western blot result shows the phosphorylation level of eIF2α in APE1WT, APE1KD and APE1OE cells after 0.5 mM H_2_O_2_ treatment. **g** Relative abundance of p-eIF2α in APE1KD and APE1OE cells compared to APE1WT is analyzed by One-way ANOVA. Mean ± SEM is shown (n = 3). **h** Representative images of immunofluorescence (IF) studies conducted in APE1KD cells transfected with APE1 WT and mutants C65S and E96A. Cells were treated with 0.5 mM H_2_O_2_ for 3 h to induce SGs. Coverslips were probed with p-eIF2α and G3BP1 antibodies to mark SGs. p-eIF2α (red) and G3BP1 (green) were merged with the nuclear stain DAPI (blue). Scale bar, 20 μm. **i** Quantification of the percentage of cells containing SGs in cells transfected with APE1 mutants relative to APE1 WT. Data represent means ± SEM of n = 3 independent replicates. **j** Representative western blot result shows the phosphorylation level of eIF2α in transfected cells after H_2_O_2_ treatment. **k** Relative abundance of p-eIF2α in transfected were quantified and analyzed by One-way ANOVA. Mean ± SEM is shown (n = 3). All experiments were independently repeated for more than 3 times. Image J software was used for the quantification of WB bands and IF images. GraphPad Prism was used to conduct statistical analysis. *p < 0.05, **p < 0.01, ***p < 0.001

Overexpression of APE1 is indicative of poor survival in cancer patients [22]. To validate this in OC, Kaplan–Meier survival analysis was performed on 1435 OC cases using the Kaplan–Meier Plotter (https://kmplot.com/analysis/). The analysis revealed a significant correlation between high APE1 mRNA levels and poor progression-free survival (PFS) of patients (*p* < 0.001; Fig. 1a). Cisplatin is the most effective drug for treating OC [49]. Based on PFS, OC patients were divided into two groups: cisplatin sensitive group (n = 12, PFS ≤ 6 months) and cisplatin resistant group (n = 13, PFS > 12 months). Immunohistochemical (IHC) staining was performed to analyze the expression profile of APE1. The results showed significantly higher APE1 expression in the cisplatin-resistant group (Fig. 1b, c).

To investigate the functional role of APE1 in OC, stable cell lines with APE1 knockdown and overexpression were established in a typical ovarian cancer cell line SKOV3. The APE1 knockdown cell line (APE1KD) was generated using the lentiCRISPR v2 plasmid (Addgene), while the APE1 overexpression cell line (APE1OE) was constructed using the pCDH-CMV vector (Addgene). The expression levels of APE1 were confirmed by quantitative MS (Supplementary Table 1) and western blotting (WB) (Supplementary Fig. 1A).

Previous studies have shown that APE1 promotes cancer cell proliferation and migration [50–52]. To validate these findings, we performed the proliferation assay to assess the impact of APE1 on cell proliferation. The results showed a significant enhancement of cell proliferation by APE1 (Supplementary Fig. 1B). We then conducted the transwell assay to evaluate the influence of APE1 on cell migration. Our results indicate that cell migration is significantly increased by APE1 (Supplementary Fig. 1C, D). To investigate how APE1 affects cellular sensitivity to drugs, we treated APE1WT, APE1KD, and APE1OE cells with methyl methanesulfonate (MMS), cisplatin, and H_2_O_2_ followed by cell viability assessment with a cell-counting kit (CCK-8, Beyotime). Notably, APE1 knockdown sensitized cells to these drugs, while APE1 overexpression substantially facilitated cell survival rates after treatment (Supplementary Fig. 1E–G). Collectively, these findings demonstrate that APE1 positively regulates cell migration, proliferation, and promotes cell survival under stress.

However, the underlying mechanism by which APE1 raises drug resistance in cells remains unclear. The formation of SGs is an essential defense mechanism cells employ under stressful conditions. However, no research has yet explored the potential link between APE1 and SGs. To investigate this possibility, we evaluated whether APE1 modulates SG formation after drug treatment. APE1WT, APE1KD, and APE1OE cells were treated with 0.5 mM H_2_O_2_ for 3 h or 250 μM cisplatin for 4 h, followed by immunofluorescent staining against SG markers phosphorylated eIF2α (p-eIF2α) and G3BP1 (Fig. 1d, Supplementary Fig. 2A). Notably, APE1OE showed a significant elevation in the percentage of SG foci-containing cells relative to APE1WT, whereas APE1 knockdown largely reduced SG formation (Fig. 1e, Supplementary Fig. 2B). To confirm these findings, western blot analysis was conducted on APE1WT, APE1KD, and APE1OE cells after treatment to assess the relative abundance of p-eIF2α (Fig. 1f, Supplementary Fig. 2C). Consistent with the IF results, the level of p-eIF2α was significantly increased by APE1 overexpression (Fig. 1g, Supplementary Fig. 2D). We then established APE1 knockdown (APE1_KD) and overexpression (APE1_OE) cell lines on another ovarian cancer cell line A2870 (Supplementary Fig. 2E) and found that APE1 also facilitated SG formation after H_2_O_2_ or cisplatin treatment (Supplementary Fig. 2F–M).

Since APE1 has multiple functions, it’s interesting to investigate which function is mainly responsible for SG regulation. Therefore, we generated APE1 mutants that are either redox-defective (C65S) or nuclease-defective (E96A). Interestingly, the mutant E96A was also reported to abolish the pri-miRNA processing function of APE1 [35]. Flag-tagged wildtype APE1 and the mutants were transfected into APE1KD cells. After SG induction, cells transfected with APE1 C65S but not E96A displayed a substantial reduction in SG formation relative to wildtype APE1 (Fig. 1h, i). This result was validated by WB analysis on p-eIF2α (Fig. 1j, k). Together, our data provide the first evidence that APE1 promotes SG formation and suggest that the redox function of APE1 is involved in SG regulation.

### APE1 interacts with YBX1 and G3BP1 and colocalizes with SGs

APE1 has been reported to interact with YBX1 in the nucleus to facilitate MDR1 expression in cells [21]. YBX1 is predicted to have two intrinsically disordered regions (IDRs), an important driver for phase separation, in its N- and C-terminus, separated by a cold-shock domain (CSD), according to IUPred3 (https://iupred2a.elte.hu/; Supplementary Fig. 3A). Over 62% of the amino acid residues in YBX1 has a prediction score over 0.8 (Supplementary Table 2), indicating an over 80% probability of these residues being part of a disordered region. This prediction implies that YBX1 is highly disordered and may undergo phase separation.

To assess the capacity of YBX1 to form biomolecular condensates in vitro, we purified monomeric enhanced GFP (mEGFP)-tagged YBX1 and performed an in vitro phase separation assay. We observed phase separation of YBX1 at protein concentration of as low as 5 μM (Supplementary Fig. 3B). For visualization of in vivo condensates, we transiently transfected 293 T cells with mEGFP-YBX1 and detected cytoplasmic condensates using live cell imaging (Supplementary Fig. 3C). Fluorescence recovery after photobleaching (FRAP) experiments on the cytoplasmic condensates revealed complete fluorescence recovery within 5 min, indicating a liquid-like behavior (Supplementary Fig. 3D). These results demonstrate that YBX1 undergoes phase separation to form liquid condensates both in vitro and in vivo, consistent with previous research [53].

Furthermore, YBX1 was identified in SGs through MS analysis [54, 55] and exhibits colocalization with SGs in U2OS cells [19]. To investigate whether YBX1 forms liquid condensates in response to SG-inducing agents in SKOV3 cells, we transfected SKOV3 cells with mEGFP-YBX1 and treated them with arsenite or H_2_O_2_. Live cell imaging of the treated cells revealed the formation of cytoplasmic condensates (Supplementary Fig. 3E). Immunofluorescence assays on arsenite or H_2_O_2_-treated SKOV3 cells confirmed the colocalization of YBX1 and G3BP1, further validating the SG localization of YBX1 in SKOV3 cells (Supplementary Fig. 3F).

**Fig. 2:**
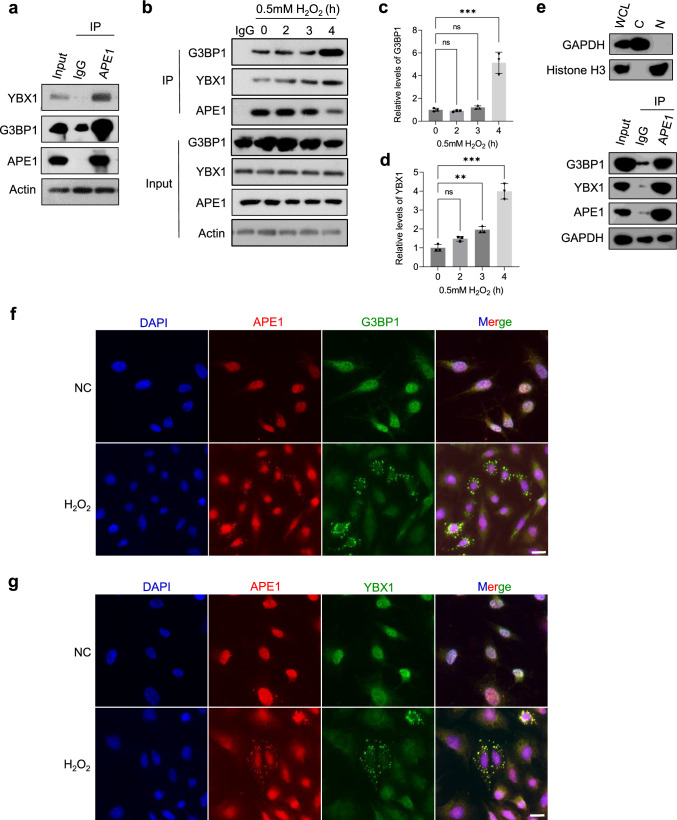
APE1 interacts with G3BP1 and YBX1 and colocalizes with SGs. **a** Immunoprecipitation (IP) was performed in SKOV3 cells against APE1. Anti-G3BP1 and anti-YBX1 antibodies were used to detect interacting G3BP1 and YBX1, respectively. **b** Co-immunoprecipitation assay conducted using anti-APE1 antibody on SKOV3 cells treated with increasing time of exposure to H_2_O_2_. Anti-G3BP1 and anti-YBX1 antibodies were used to detect interacting G3BP1 and YBX1, respectively. **c**, **d** One-way ANOVA was performed to analyze interacting G3BP1 (**c**) and YBX1 (**d**). **p < 0.01, ***p < 0.001. **e** Co-immunoprecipitation assay was performed using the cytoplasmic fraction obtained by subcellular fractionation. Anti-GAPDH and anti-Histone H3 antibodies were used to assess the purity of the fractions. Anti-APE1 antibody was used immunoprecipitate APE1. Anti-G3BP1 and anti-YBX1 antibodies were used to detect interactions. **f** Representative images of immunofluorescence (IF) studies showing the colocalization of APE1 and G3BP1. Coverslips were probed with anti-APE1 (red) and anti-G3BP1 (green) antibodies, which were merged with the nuclear stain DAPI (blue). Zoom represents magnified inset. Scale bar, 20 μm. **g** Representative images of immunofluorescence (IF) studies showing the colocalization of APE1 and YBX1. Coverslips were probed with anti-APE1 (red) and anti-YBX1 (green) antibodies, which were merged with the nuclear stain DAPI (blue)

Therefore, it is intriguing to explore whether APE1 displays SG localization and interacts with SG proteins such as YBX1 in SGs. To address these questions, we first investigated the interaction between APE1 and YBX1 or the central SG protein G3BP1. Co-immunoprecipitation experiments on SKOV3 cells targeting APE1 detected strong interaction between APE1 and YBX1 as well as G3BP1 (Fig. 2a). Furthermore, significant increases in interacting YBX1 and G3BP1 were observed upon treatment (Fig. 2b–d), particularly after 4 h of treatment. Additionally, we performed subcellular fractionation to isolate the cytoplasm of cells. Co-IP was then conducted on the cytoplasmic fraction against APE1 and detected robust interactions (Fig. 2e). To visualize their cellular localization, we performed immunofluorescence staining on H_2_O_2_-treated and untreated SKOV3 cells, targeting APE1 and G3BP1 (Fig. 2f) or APE1 and YBX1 (Fig. 2g). Cytoplasmic colocalization was observed between APE1 and YBX1 as well as G3BP1. Our findings show that APE1 can be recruited to SGs under stress.

### APE1 alters cellular phospho-landscape especially the phosphoprofile of SG proteins

Despite being implied in several kinase signaling pathways [36, 37], the impact of APE1 on the phosphoproteome of cells remains poorly understood. As SGs are regularly modulated by phosphorylation, we are interested in whether APE1 regulates SG formation through phorphorylation. We conducted label-free quantitative phosphoproteomic and global proteomic analysis on APE1WT, APE1KD, and APE1OE cells. We followed the high-sensitivity EasyPhos workflow [43] with minor modifications to prepare samples for MS analysis (Supplementary Fig. 4A). The data were acquired using an ultra-high performance liquid chromatography-Orbitrap Fusion Tribrid mass spectrometer and analyzed by Proteome discoverer 2.5 software.

Overall, 9200 distinct localized phosphorylated sites in 8382 phosphopeptides on 2916 different proteins were identified, where 2181 phosphosites quantified were distinctly modulated by APE1 at a false discovery rate (FDR) of 5% (Supplementary Table 3). The number of phosphopeptides containing one (1P), two (2P), or three (3P) phosphate groups are 5648, 3422 and 425, respectively (Supplementary Fig. 4B). Serine, threonine and tyrosine residues accounted for 7903, 1211 and 76 of the identified phosphosites respectively (Supplementary Fig. 4C).

**Fig. 3:**
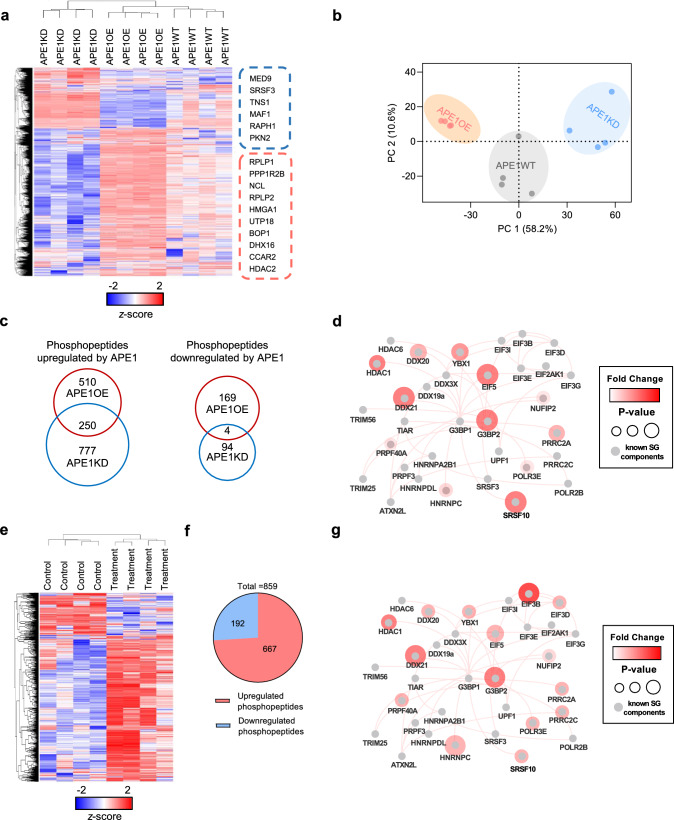
APE1 alters cellular phospho-landscape and phospho-profile of SG proteins. **a** Unsupervised hierarchical clustering of phosphopeptides of APE1WT, APE1KD and APE1OE. Top regulated phosphoproteins were listed on the right. Blue box, phosphoproteins negatively regulated by APE1; Red box, phosphoproteins positively regulated by APE1. **b** Principal Component Analysis (PCA) of the phosphoproteome showed small variation between replicates and differences among cell lines. **c** Left Venn diagram shows phosphopeptides differentially and simultaneously upregulated by APE1 in APE1KD (*p* < 0.01, phosphopeptide abundance: APE1KD/APE1WT < 0.5) and APE1OE (*p* < 0.01, phosphopeptide abundance: APE1OE/APE1WT > 2). Right Venn diagram shows phosphopeptides differentially and simultaneously downregulated by APE1 in APE1KD (*p* < 0.01, phosphopeptide abundance: APE1KD/APE1WT > 2) and APE1OE (*p* < 0.01, phosphopeptide abundance: APE1OE/APE1WT < 0.5). **d** Core SG proteins in the SG PPI network modulated by APE1. The gray nodes are known proteins involved in SGs. The red nodes are phosphoproteins distinctly modulated by APE1. The color and size of the nodes represent the fold change and *p*-value of the phosphorylation status of the protein. **e** Unsupervised hierarchical clustering of phosphopeptides from untreated and H_2_O_2_-treated SKOV3 cells. **f** A total of 1239 phosphopeptides are significantly regulated upon treatment (*p* < 0.01). Among them, 322 phosphopeptides are downregulated and 917 phosphopeptides are upregulated after H_2_O_2_ treatment. **g** Phosphoprofile alteration of core SG proteins upon H_2_O_2_ treatment. The gray nodes are known proteins involved in SGs. The red nodes are phosphoproteins upregulated after H_2_O_2_ treatment. The color and size of the nodes represent the fold change and *p*-value of the phosphorylation status of the proteins

Unsupervised hierarchical clustering of phosphopeptides was done across all samples with top upregulated (red box) and downregulated (blue box) phosphoproteins listed on the right (Fig. 3a). Principal component analysis (PCA) displayed small variation within group and distinct differences among groups, indicating a critical impact of APE1 on cellular phosphoproteome (Fig. 3b). We then analyzed phosphorylation of proteins simultaneously or differentially up- and downregulated by APE1 in APE1KD and APE1OE cells. Notably, 250 phosphopeptides on 188 proteins were distinctively upregulated (*p* < 0.01, phosphopeptides abundance: APE1OE/APE1WT > 2 and APE1KD/APE1WT < 0.5) by APE1 in both APE1KD and APE1OE (Supplementary 4, Fig. 3c). However, only 4 phosphopeptides on 4 proteins were significantly downregulated (*p* < 0.01, phosphopeptides abundance: APE1OE/APE1WT < 0.5 and APE1KD/APE1WT > 2) by APE1 in both APE1KD and APE1OE (Supplementary Table 4, Fig. 3c). These findings demonstrate that APE1 generally promotes phosphorylation in cells. To gain insights into the functional implications of the significantly regulated phosphoproteins, we performed gene ontology (GO) analysis. The analysis revealed highly enriched cellular processes, including regulation of mRNA metabolic process, RNA splicing, spliceosomal complex, and RNA polymerase binding (Supplementary Fig. 3D). In contrast to the substantial changes observed in the phosphoproteome, the global proteome exhibited limited alterations by APE1. Out of the 4751 proteins identified and quantified, only 29 proteins showed significant regulation by APE1 (*p* < 0.05, unique peptides > 1, protein abundance: APE1OE/APE1KD > 2 or < 0.5) (Supplementary Table 5). These findings indicate that APE1 primarily affects the phosphoproteome rather than the overall proteome of cells.

Phosphorylation is one of the most extensively studied PTMs that modulate SGs. To assess whether APE1 modulates SG formation through phosphorylation, we curated SG proteins from the RNA Granule Database (http://rnagranuledb.lunenfeld.ca/) and screened for differentially regulated SG proteins. We identified a total of 37 SG proteins that were significantly regulated by APE1 (*p* < 0.01, abundance of upregulated phosphopeptides: APE1OE/APE1WT > 2 and APE1KD/APE1WT < 0.5; *p* < 0.01, abundance of downregulated phosphopeptides: APE1OE/APE1WT < 0.5 and APE1KD/APE1WT > 2), including core SG components G3BP2, EIF5, DDX20 and DDX21 (Supplementary Table 6). For example, phosphorylation of G3BP2 at S149 was upregulated by APE1. Meanwhile, double phosphorylation of EIF5 at S389 and S390 was enhanced by APE1. Importantly, these phosphopeptides are mostly upregulated by APE1, suggesting that APE1 plays a significant role in the upregulation of phosphorylation of SGs proteins. The protein–protein interaction (PPI) network visualizes the interacting core SG proteins [56–58], with the phosphoproteins significantly modulated by APE1 denoted, including YBX1, G3BP2, and EIF5 (Fig. 3d). These results demonstrate the important regulatory role of APE1 in SG dynamics.

To investigate how the phosphoprofile of SG proteins modifies after stress, we performed quantitative phosphoproteomic analysis on H_2_O_2_-treated and untreated SKOV3 cells (Supplementary Table 7). Unsupervised hierarchical clustering of phosphopeptides was done across all samples (Fig. 3e). A total of 859 phosphopeptides on 592 proteins are distinctly modulated after H_2_O_2_ treatment. Among them, 667 phosphopeptides were significantly upregulated (*p* < 0.01, phosphopeptides abundance: Treatment/Control > 2) after treatment, whereas 192 phosphopeptides were significantly downregulated (*p* < 0.01, phosphopeptides abundance: Treatment/Control < 0.5; Fig. 3f; Supplementary Table 7). Importantly, phosphorylation of 162 SG proteins were distinctly modulated upon treatment (Supplementary Table 7). The PPI network of core SG proteins under treatment is shown (Fig. 3g). A substantial overlap (73%) of the phosphoprofile alteration in core SG proteins was observed between drug treatment and APE1 overexpression, including YBX1, DDX20, DDX21, G3BP2 and EIF5. Such overlap implies that APE1 may prepare cells before the assaults by preshaping the phosphoprofile of SG proteins. Our findings reveal a novel regulatory role of APE1 in promoting chemoresistance in cancer.

To decipher how APE1 exerts such an impact on the phosphoproteome of cells, we analyzed the kinase signaling pathways. Unsupervised hierarchical clustering of 276 significantly regulated (*p* < 0.01, phosphopeptide abundance: APE1OE/APE1KD > 2 or < 0.5) phosphopeptides on 160 proteins involved in kinase signaling pathways was performed. Top regulated protein kinases includes AKT1, MAP2K2, and CDK12 (Supplementary Fig. 5A, Supplementary Table 6). Out of the 160 proteins involved in kinase signaling pathways, 62 are protein kinases (Supplementary Fig. 5B, Supplementary Table 6). GO enrichment analysis of differentially phosphorylated proteins involved in kinase signaling pathways revealed significant regulation in pathways such as JAK-STAT signaling pathway, AMPK signaling pathway, and ErbB signaling pathway (Supplementary Fig. 5C). In addition, phosphorylation of several phosphatases are found to be distinctively regulated by APE1, including PPP1R2, PPP1R12A, PTPN23, etc. (Supplementary Table 6). Modulation of protein kinases, phosphatases and signaling pathways may explain the distinctive alterations in the cellular phosphoprofile caused by APE1.

### APE1 facilitates phosphorylation of YBX1 at S174 and S176 to enhance SG formation

**Fig. 4:**
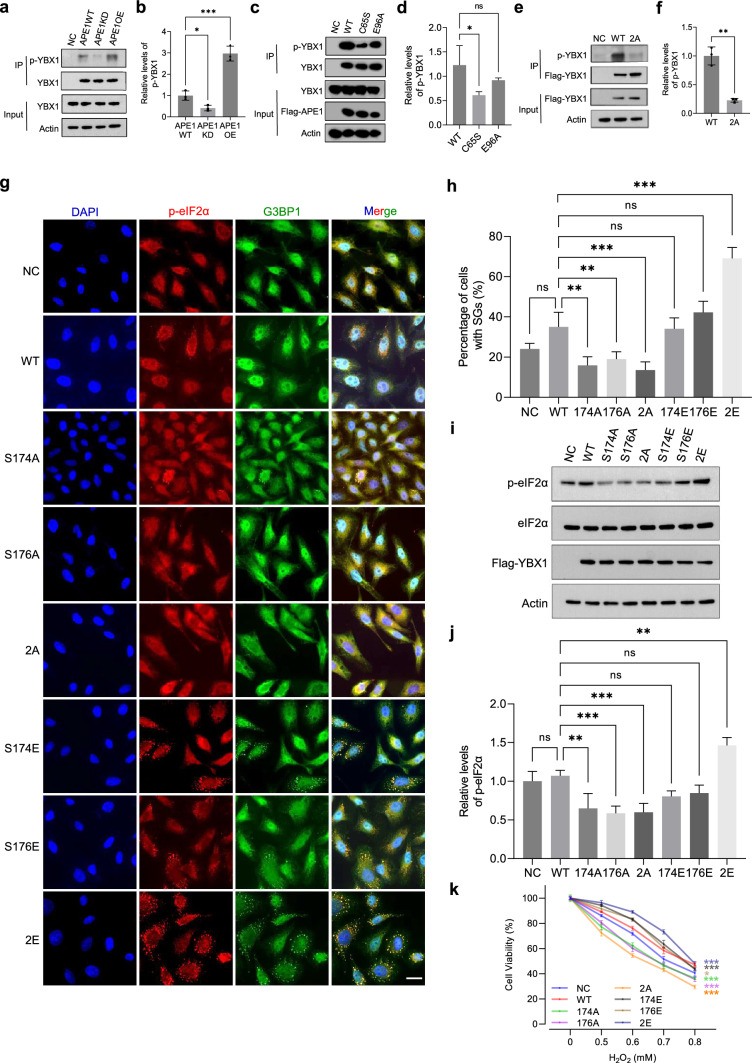

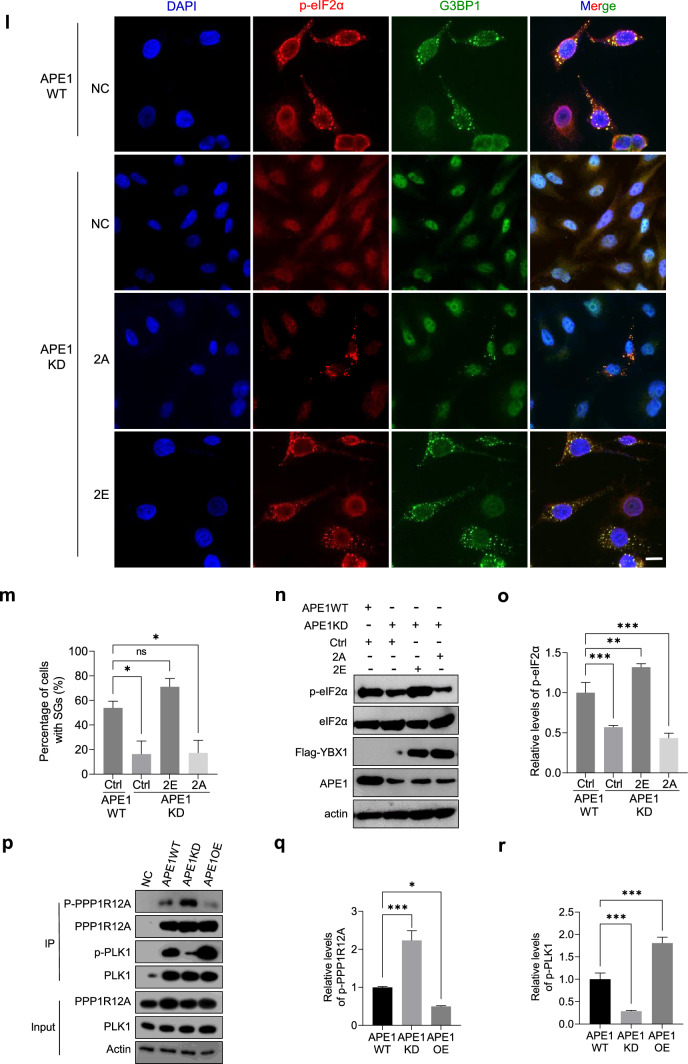
APE1 enhances phosphorylation of YBX1 at S174 and S176 to promote SG formation and cell survival. **a** Immunoprecipitation (IP) was performed in APE1WT, APE1KD and APE1OE cells against YBX1. Anti-phospho-serine antibody was used to evaluate the phosphorylation level of YBX1. YBX1 and actin in the whole cell lysate (WCL) were shown in the input. **b** Relative abundance of p-YBX1 was quantified and analyzed using one-way ANOVA. Mean ± SEM is shown (n = 3). **c** Immunoprecipitation was performed on APE1KD cells transiently transfected with Flag-tagged APE1 WT and mutants against Flag-tag. Abundance of phosphorylated YBX1 (p-YBX1) was probed using anti-phosphoserine antibody. Input shows the level of actin and exogenous Flag-APE1 in the WCL. **d** Relative abundance of p-YBX1 was quantified and analyzed by One-way ANOVA. Mean ± SEM is shown (n = 3). **e** Immunoprecipitation was performed on SKOV3 cells transiently transfected with Flag-tagged YBX1 WT and 2A against Flag-tag. Abundance of phosphorylated YBX1 (p-YBX1) was probed using anti-phospho-serine antibody. Input shows the level of actin and exogenous Flag-YBX1 in the WCL. **f** Relative abundance of p-YBX1 was quantified and analyzed by One-way ANOVA. Mean ± SEM is shown (n = 3). **g** Representative images of immunofluorescence (IF) studies conducted on SKOV3 cells transiently transfected with Flag-tagged YBX1 WT and the indicated mutants followed by H_2_O_2_ treatment. Coverslips were probed with p-eIF2α antibody and G3BP1 antibody for the visualization of cytoplasmic SGs. Scale bar, 20μm. **h** The number of cells containing SGs was recorded and analyzed by One-way ANOVA using YBX1 WT as control. Mean ± SEM is shown (n = 3). **i** Representative western blot shows the relative abundance of p-eIF2α in SKOV3 cells transfected with YBX1 WT and the indicated mutants after H_2_O_2_ treatment. **j** Relative abundance of p-eIF2α was statistically analyzed using YBX1 WT as control. Mean ± SEM is shown (n = 3). **k** Cell viability was assessed on SKOV3 cells transfected with YBX1 WT and mutants after H_2_O_2_ treatment using a CCK-8 kit. Mean ± SEM is shown (n = 4). **l** Representative images of immunofluorescence (IF) studies conducted in APE1WT and APE1KD cells transfected with empty vector (NC) or YBX1 mutants (2A or 2E). Cells were treated with 0.5 mM H_2_O_2_ for 3 h. Coverslips were probed with p-eIF2α and G3BP1 antibodies. p-eIF2α (red) and G3BP1 (green) were merged with the nuclear stain DAPI (blue). Scale bar, 20 μm. **m** Quantification of the percentage of cells with SGs in transfected APE1KD and APE1WT cells. Means ± SEM is shown (n = 3). **n** Representative western blot result shows the level of p-eIF2α in transfected APE1WT and APE1KD cells after 0.5 mM H_2_O_2_ treatment. **o** Relative abundance of p-eIF2α in transfected APE1WT and APE1KD cells was analyzed by One-way ANOVA. Mean ± SEM is shown (n = 3). **p** Immunoprecipitation was performed in APE1WT, APE1KD and APE1OE cells against PPP1R12A or PLK1. Anti-phosphoserine antibody was used to evaluate the phosphorylation level. Proteins in the whole cell lysate (WCL) were shown in the input.** q** Relative abundance of p-PPP1R12A was quantified and analyzed using one-way ANOVA. Mean ± SEM is shown (n = 3).** r** Relative abundance of p-PLK1 was quantified and analyzed using one-way ANOVA. Mean ± SEM is shown (n = 3). All experiments were independently repeated for more than 3 times. Image J software was used for the quantification of WB bands and IF images. GraphPad Prism was used to conduct statistical analysis. *p < 0.05, **p < 0.01, ***p < 0.001

Previous studies have identified YBX1 as a target of phosphorylation by MS [59, 60], with multiple phosphorylation sites clustered in the N-terminal of its second IDR (Supplementary Fig. 6A). Our findings show that double phosphorylation of YBX1 at S174 and S176 is significantly upregulated by APE1 (Supplementary Fig. 6B). Immunoprecipitation assay was performed to confirm the phosphorylation level of YBX1 in APE1WT, APE1KD and APE1OE cells. A threefold increase in the abundance of phosphorylated YBX1 was detected in APE1OE relative to APE1WT, while a 60% reduction was observed in APE1KD (Fig. 4a, b), consistent with the MS analysis. We also assessed YBX1 phosphorylation in APE1KD cells transfected with APE1 functional mutants (Fig. 4c, d). The result showed that C65S led to a substantial decrease in p-YBX1 level, implying that the redox function of APE1 plays the main role in phosphorylation regulation. To further validate the phosphosites of YBX1, we constructed pcDNA 3.1 plasmids containing Flag-tagged wild-type YBX1 (WT) and the double mutation from serine to alanine (S174/176A, 2A) to mimic the constitutively dephosphorylated status. Immunoprecipitation was performed on SKOV3 cells transiently transfected with Flag-tagged YBX1 WT and 2A plasmids using anti-flag M2 beads (Sigma-Aldrich). We found that the S to A double mutation caused a significant reduction in the level of phosphorylated YBX1 (Fig. 4e, f). These results indicate that APE1 enhances YBX1 phosphorylation and that S174 and S176 are the primary phosphosites.

To evaluate the functional significance of YBX1 phosphorylation at S174 and S176, we created phosphomutants to mimic hyperphosphorylation (serine to glutamic acid, S to E) and hypophosphorylation (serine to alanine, S to A). These include double mutation S174/176A (2A) and S174/176E (2E) and single mutation S174A, S176A, S174E and S176E (Supplementary Fig. 6C). SKOV3 cells were transfected with Flag-tagged YBX1 WT and each mutant, followed by H_2_O_2_ treatment to induce SG formation. Notably, 2E resulted in a much higher percentage of cells containing SGs, whereas 2A substaintially reduced cellular SG formation (Fig. 4g, h). Single mutations displayed more moderate effects on SG formation compared to double phosphorylation (Fig. 4g, h). In consistency with IF results, cellular level of p-eIF2α was substaintially elevated in SKOV3 cells transfected with 2E, while transfection of 2A plasmids led to a substantial decrease in p-eIF2α. Similarly, single mutations to E had limited impact on the level of p-eIF2α (Fig. 4i, j). To determine the impact of YBX1 phosphorylation on cell survival, we performed the cell viability assay with YBX1 WT and mutants. SKOV3 cells transfected with 2E exhibited greater resilience under H_2_O_2_ treatment, whereas 2A sensitized cells to treatment. Single phosphorylation partially contributed to cell survival under H_2_O_2_ treatment (Fig. 4k). To validate whether YBX1 phosphorylation compensates APE1 depletion and recovers SG formation, we transfected APE1KD cells with YBX1 phosphomutants 2A or 2E followed by H_2_O_2_ treatment to induce SG formation. Our data demonstrate that 2E significantly increased cellular SG formation compared to 2A (Fig. 4l, m), suggesting that phosphorylation of YBX1 at S174 and S176 restored SG formation impaired by APE1 depletion. In agreement with the IF results, transient expression of 2E, rather than 2A, recovered the diminished p-eIF2α level in APE1KD cells (Fig. 4n, o). These findings indicate that APE1 promotes YBX1 phosphorylation at S174 and S176 to enhance SG formation and cell survival under adverse conditions.

Recently, a study published by Li et al*.* demonstrates that Polo-kinase 1 (PLK1) directly binds to YBX1 and phosphorylates YBX1 at S174 and S176 [61]. Moreover, our phosphoproteomic data showed that APE1 significantly downregulated phosphorylation of protein phosphatase 1 regulatory subunit 12A (PPP1R12A, relative abundance: APE1OE/APE1KD = 0.076, *p* = 0.043), whereas the phosphatase PPP1R12A has been shown to dephosphorylate PLK1 [62]. We speculate that APE1 may promote YBX1 phosphorylation via downregulating PPP1R12A phosphorylation, leading to increased PLK1 phosphorylation, which in turn phosphorylates YBX1 at S174 and S176. Therefore, we performed immunoprecipitation assay on APE1WT, APE1KD and APE1OE cells against PPP1R12A and PLK1. APE1OE cells displayed a significantly lower level of phosphorylated PPP1R12A and a substantially elevated level of phosphorylated PLK1, compared to APE1WT, while a significant increase in phosphorylated PPP1R12A and a notable reduction of phosphorylated PLK1 were observed in APE1KD cells (Fig. 4p–r). To further evaluate the impact of YBX1 phosphorylation, we performed in vitro phosphorylation assay followed by phase separation experiment with freshly purified mCherry-APE1, mEGFP-YBX1, PLK1 and cell lysate. The results show that PLK1 phosphorylated YBX1 in vitro (Supplementary Fig. 6D). Colocalization of APE1 and YBX1 manifested after phosphorylation assay with PLK1 (Supplementary Fig. 6E). Importantly, phosphorylation of YBX1 significantly enhanced droplet formation at the concentration of 10 μM (Supplementary Fig. 6E). Our data demonstrate that phosphorylation of YBX1 by PLK1 facilitates phase separation of YBX1 and increases the colocalization of APE1 and YBX1. Taken together, our findings demonstrate that APE1 may promote YBX1 phosphorylation via PPP1R12A and PLK1 to enhance SG formation.

## Discussion

Cancer cells are constantly under adverse conditions in the tumor microenvironment. Responding and adapting to stress is vital in cancer development and anticancer therapies [63]. The formation of SGs can protect cancer cells from apoptosis and induce resistance against anti-cancer drugs and radiation treatment, making SGs promising targets for cancer treatment [64]. Previous studies have shown that APE1 reduces patient survival by promoting multiple malignant properties of cancer, including drug resistance [24, 25, 65]. Yet how APE1 facilitates cell survival under drug treatment remains unclear. In this study, we investigated the possibility of regulation of SGs by APE1 and its underlying mechanisms.

To explore how APE1 promotes cancer cell survival under stress, we established stable cell lines with APE1 overexpression and knockdown using SKOV3. APE1 knockdown sensitized SKOV3 cells to MMS, cisplatin and H_2_O_2_, while APE1 overexpression significantly enhanced cell survival rate after treatment, in consistent with previous studies [26, 66, 67]. To investigate whether APE1 influences SG formation in cells, we treated these cell lines with H_2_O_2_ or cisplatin and probed for SG formation. Our data demonstrate that APE1 significantly facilitate SG formation in cells. Moreover, we created functional mutants C65S and E96A of APE1 and found that the redox function of APE1 plays the major role in SG regulation. In addition, we demonstrate that both YBX1 and G3BP1 are interaction partners of APE1 in the cytoplasm and their interaction increased after SG-induction. Moreover, APE1 exhibits SG localization though its role in SG is currently unknown. Our data show that APE1 not only colocalizes with SG but also promotes cell survival via modulation of SG formation.

As regulation of SGs by phosphorylation has been widely reported [16–18, 54, 61], it would be interesting to see whether APE1 regulates phosphorylation of SG proteins. Therefore, we performed label-free quantitative phosphoproteomics in APE1WT, APE1KD and APE1OE cells. Notably, APE1 significantly alters the phospho-landscape of SKOV3, suggesting a novel and intricate network of proteins under the regulation of APE1 presumably through phosphorylation. A total of 8382 phosphopeptides on 2916 different proteins were identified, among which 2181 phosphosites quantified were distinctly regulated. Most importantly, phosphorylation of 37 SG related proteins is distinctively regulated by APE1, including G3BP2, DDX20, DDX21, EIF5, YBX1, etc. Both G3BP1 and G3BP2 contribute partially to SG formation, while the overexpression of any of the two induces SG formation without stress stimuli [68]. Our data show that phosphorylation of G3BP2 at S149 was upregulated by APE1. While phosphorylation of S149 in IDR1 of G3BP1 was shown to be a negative regulator of SG assembly as dephosphorylation at S149 stimulates the multimerization of G3BP1and facilitates SG formation [17, 57], more research is needed to elucidate the role of G3BP2 phosphorylation at S149. Translation initiation factors such as EIF2, EIF3 and EIF5 are essential components in SGs. SGs can be divided into 3 subtypes according to the translation initiation factors and RNA-binding proteins contained [69, 70]. EIF5 may be present in type II and type III SGs [69]. Our data showed that APE1 significantly enhanced phosphorylation of EIF5 at S389 and S390. Casein kinase 2 (CK2) was found to phosphorylate EIF5 at S389 and S390, which is associated with cell cycle progression [71]. Moreover, phosphorylation by CK2 significantly increases affinity between EIF5 and EIF2 [8]. While phosphorylation of EIF2α is essential for efficient SG formation [5, 72], the effect of EIF5 phosphorylation on SG dynamics remains unclear. Members of the family of RNA-dependent DEAD-box ATPases (DDXs) regulate RNA-containing phase-separated organelles in cells [73]. Our data revealed that APE1 promoted phosphorylation of both DDX20 and DDX21. Collectively, APE1 significantly alters the phosphoprofile of essential SG proteins. Further investigations are needed to dissect how APE1 modulates these phosphorylation events and elucidate their effects on SG dynamics and downstream mRNA translation.

Moreover, a systemic phosphoprofiling of the SG proteins under stress is lacking. To decipher how the cellular phosphoproteome reacts to stress, we conducted phosphoproteomic analysis on H_2_O_2_ treated SKOV3 cells. Our data revealed 162 SG proteins that are specifically modulated under stress conditions. Interestingly, we observed a significant overlap between APE1-promoted and stress-induced phosphorylation events in the core SG protein interaction network. These proteins include YBX1, EIF5, G3BP2, DDX20, DDX21, HDAC1, HNRNPC, SRSF10, etc. This finding suggests that APE1 proactively prepares cells for potential assaults through reshaping the phospho-profile of SG components to enhance SG formation.

Recent studies have revealed the involvement of APE1 in several kinase signaling pathways [36, 39, 74]. Yet the kinases, phosphatases and kinase signaling pathways involved remain unknown. To explore how APE1 promotes phosphorylation of SG proteins, we analyzed proteins engaged in protein kinase pathways and found phosphorylation of 62 kinases that are significantly modified by APE1, including AKT1, AKT2, MAP2K1, ROCK1, etc. Several phosphatases also showed significant regulation by APE1 such as PPP1R2 and PPP1R12A. Additionally, our data reveal enriched kinase signaling pathways including JAK-STAT, AMPK, ErbB, CAMP, MTOR and PI3K-Akt signaling pathways. These signaling pathways also participate in cellular stress response [75–79]. Notably, PI3K and MAPK/p38 have been established as pro-SG-kinases which act through the metabolic master regulator mTORC1 to stimulate stress granule assembly [80]. Our findings suggest that APE1 may promote SG formation by modulating kinases and phosphatases and activating kinase signaling pathways.

Among these significantly regulated SG-related proteins, YBX1 is of special interest to us because (i) YBX1 has been reported to interact with APE1 to promote drug resistance in cells; (ii) YBX1 modulates SG formation via upregulation of G3BP1. Our MS data showed upregulation of YBX1 phosphorylation at S174 and S176 in APE1OE cells as well as in drug treated SKOV3 cells. To validate whether APE1 modulates p-YBX1, we conducted immunoprecipitation of endogenous YBX1 in APE1WT, APE1KD, and APE1OE cells and found that APE1 promotes the cellular level of p-YBX1. The modified sites were further validated by transfecting SKOV3 cells with YBX1 WT and 2A plasmids, followed by immunoprecipitation of exogenous YBX1 and assessment of p-YBX1 levels. Our results reveal that S174 and S176 are the major phosphosites of YBX1 regulated by APE1. To evaluate the effect of YBX1 phosphorylation on stress response, we constructed single and double mutations to mimic hypo- (S to A) and hyperphosphorylated (S to E) YBX1. We demonstrate that the YBX1 phosphomimic 2E significantly promotes SG formation and cell survival, whereas 2A reduced SG formation and sensitized cells to drug treatment. Furthermore, expressing YBX1 2E in APE1KD cells recovered SG formation and cellular resistance to drugs that were impaired by APE1 depletion. To investigate how APE1 regulates YBX1 phosphorylation, we found that APE1 downregulated phosphorylation of the phosphatase PPP1R12A which dephosphorylates PLK1, the kinase of YBX1, so as to activate PLK1 to phosphorylate YBX1. Moreover, we found phosphorylation of YBX1 by PLK1 enhanced phase separation capacity of YBX1 and facilitated colocalization of APE1 and YBX1. Together, we demonstrate the important role YBX1 phosphorylation played in SG formation and elucidate how APE1 exerts the regulatory function through kinases and phosphatases to modulate SG dynamics.

**Fig. 5:**
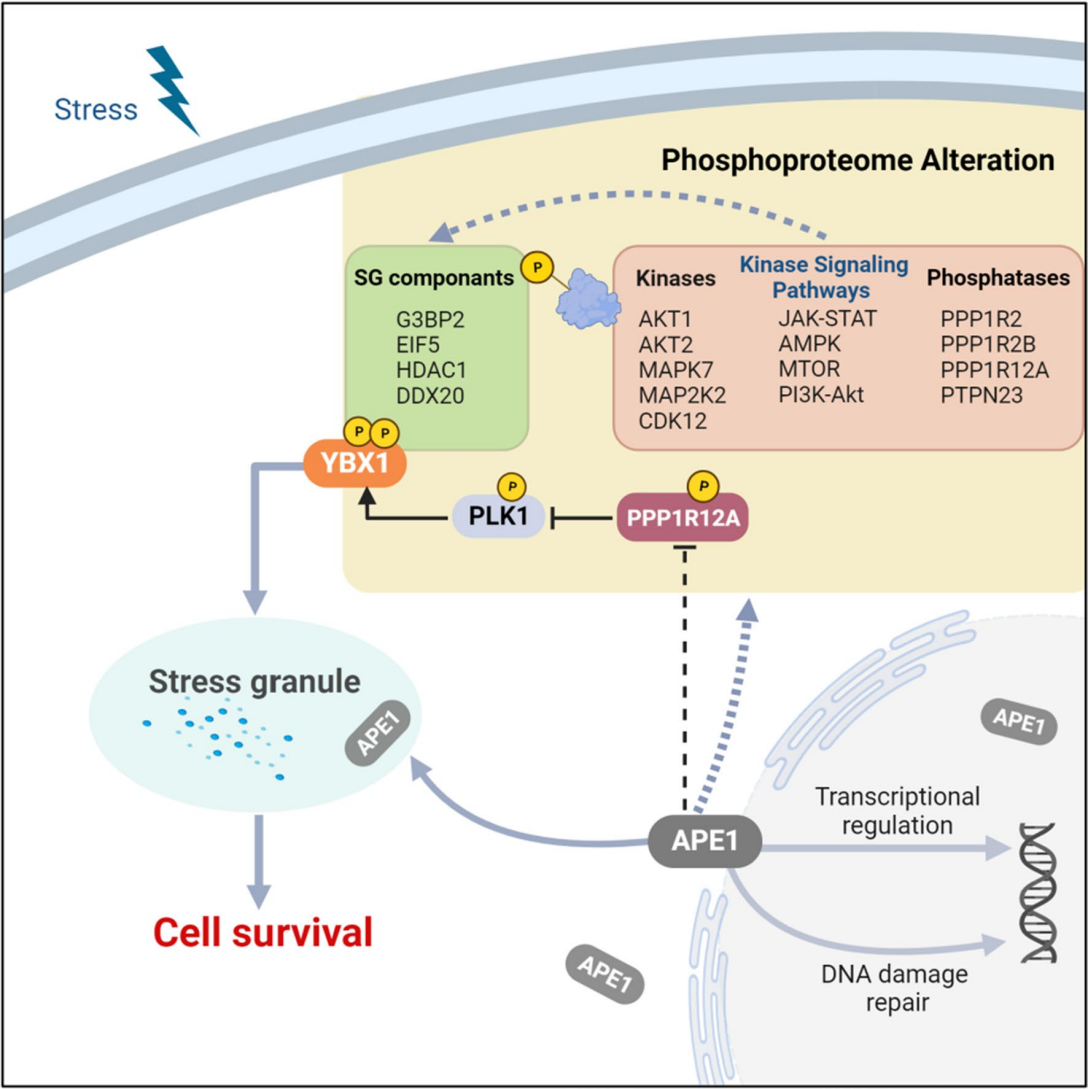
Model for SG regulation by APE1. We propose a model for SG regulation by APE1. In conjunction with its two main roles in BER and transcriptional regulation, APE1 profoundly affects the phosphoproteome of cells, including kinase signaling pathways, protein kinases such as AKT1, AKT2 and MAPK14 and phosphatases like PPP1R12A and PPP1R2 (red box). Modulation of these pathways in turn significantly alters the phosphoprofile of SG proteins (green box). Among these, APE1 downregulated phosphorylation of PPP1R12A to promote PLK1 phosphorylation which phosphorylates YBX1 at S174 and S176 to enhance SG formation and cell survival. It is worth noting that APE1 also shows SG localization

Our study unveils APE1 as a modulator of SGs and sheds light on its role in modulating the cellular phosphoproteome. We demonstrate that APE1 not only colocalizes with SGs but also promotes SG formation by modulating phosphorylation of SG proteins. Specifically, we found that APE1 promoted YBX1 phosphorylation on S174 and S176 to enhance SG formation by modulating PPP1R12A and PLK1. Thereby, we propose a model in which APE1 promotes SG formation by modulating kinases, phophatases and kinase signaling pathways to influence the subsequent phosphorylation of SG proteins, especially YBX1 to facilitate cell survival (Fig. 5).

These findings expand our knowledge in the mechanisms of how APE1 functions to safeguard cells against environmental assaults and how it contributes to chemoresistance in cancer. Interestingly, accumulating evidence are showing that APE1, an essential DNA damage repair protein, is involved in various non-DNA repair processes, especially in RNA metabolism and processing, such as rRNA quality control in nucleoli [81], miRNA processing [35] and RNA splicing [38]. In this work, we provided the first evidence that APE1 colocalizes with SGs. Since SGs are essential for RNA processing in face of stress, it would be interesting to explore the interplay between APE1 and SGs and to investigate the possible alterations in RNA processing processes in SGs in the future.

## Conclusions

In conclusion, our study reveals a novel regulatory role of APE1 in SG regulation. Our research shows that APE1 not only interacts with YBX1 and G3BP1 and colocalizes with SGs but is also a *bona fide* SG regulator. APE1 modifies the cellular phosphoproteome, especially the phosphoprofile of SG proteins to facilitate SG formation. In particular, APE1 promotes YBX1 dual-phosphorylation at S174 and S176 by modulating PPP1R12A and PLK1 to enhance SG formation and cancer cell survival. Collectively, our findings suggest that APE1 and YBX1 may serve as potential targets in future therapeutic strategies for cancer and other SG-related diseases.

### Supplementary Information

Below is the link to the electronic supplementary material.

Supplementary file 1 (XLSX 20302 KB)

Supplementary file 2 (XLSX 19 KB)

Supplementary file 3 (XLSX 5526 KB)

Supplementary file 4 (XLSX 2858 KB)

Supplementary file 5 (XLSX 26 KB)

Supplementary file 6 (XLSX 373 KB)

Supplementary file 7 (XLSX 4904 KB)

Supplementary file 8 (PDF 1246 KB)

## Data Availability

The data from this study can be accessed via ProteomeXchange with the identifier PXD040041.
