# Supplementary material for: Apurinic/apyrimidinic endodeoxyribonuclease 1 (APE1) promotes stress granule formation via YBX1 phosphorylation in ovarian cancer
Source: Cell Mol Life Sci. 2024 Mar 4;81(1):113. doi: 10.1007/s00018-023-05086-y (PMC10912283; doi:10.1007/s00018-023-05086-y)
Supplement: Supplementary file 8 — (PDF 1246 KB) [file 18_2023_5086_MOESM8_ESM.pdf]

Apurinic/apyrimidinic endodeoxyribonuclease 1 (APE1) promotes stress granule formation via YBX1 phosphorylation in ovarian cancer

Shuyu Mao[1], Chong Xie[2], Yufeng Liu[1], Ye Zhao[1], Mengxia Li[3], Han Gao[3], Yue Xiao[1], Yongkang Zou[2], Zhiguo Zheng[4], Ya Gao[1], Juan Xie[2], Bing Tian[1], Liangyan Wang[1], Yuejin Hua[1,] and Hong Xu[1, *]*

[1] MOE Key Laboratory of Biosystems Homeostasis and Protection, Institute of Biophysics, College of Life Science, Zhejiang University, Hangzhou, China.
[2]Shenzhen Bay Laboratory, Chinese Academy of Sciences, Shenzhen, Guangzhou, China
[3]Department of Cancer Center, Daping Hospital, Army Medical Univeristy, Chongqing, China
[4]The Cancer Hospital of the University of Chinese Academy of Sciences (Zhejinag Cancer Hospital), Institute of Basic Medicine and Cancer (IBMC), Chinses academy of Sciences, Hangzhou,China

Corresponding email address: xuhong1685@163.com

Submitted to ***Cellular and Molecular Life Sciences***

Supplementary Fig. 1

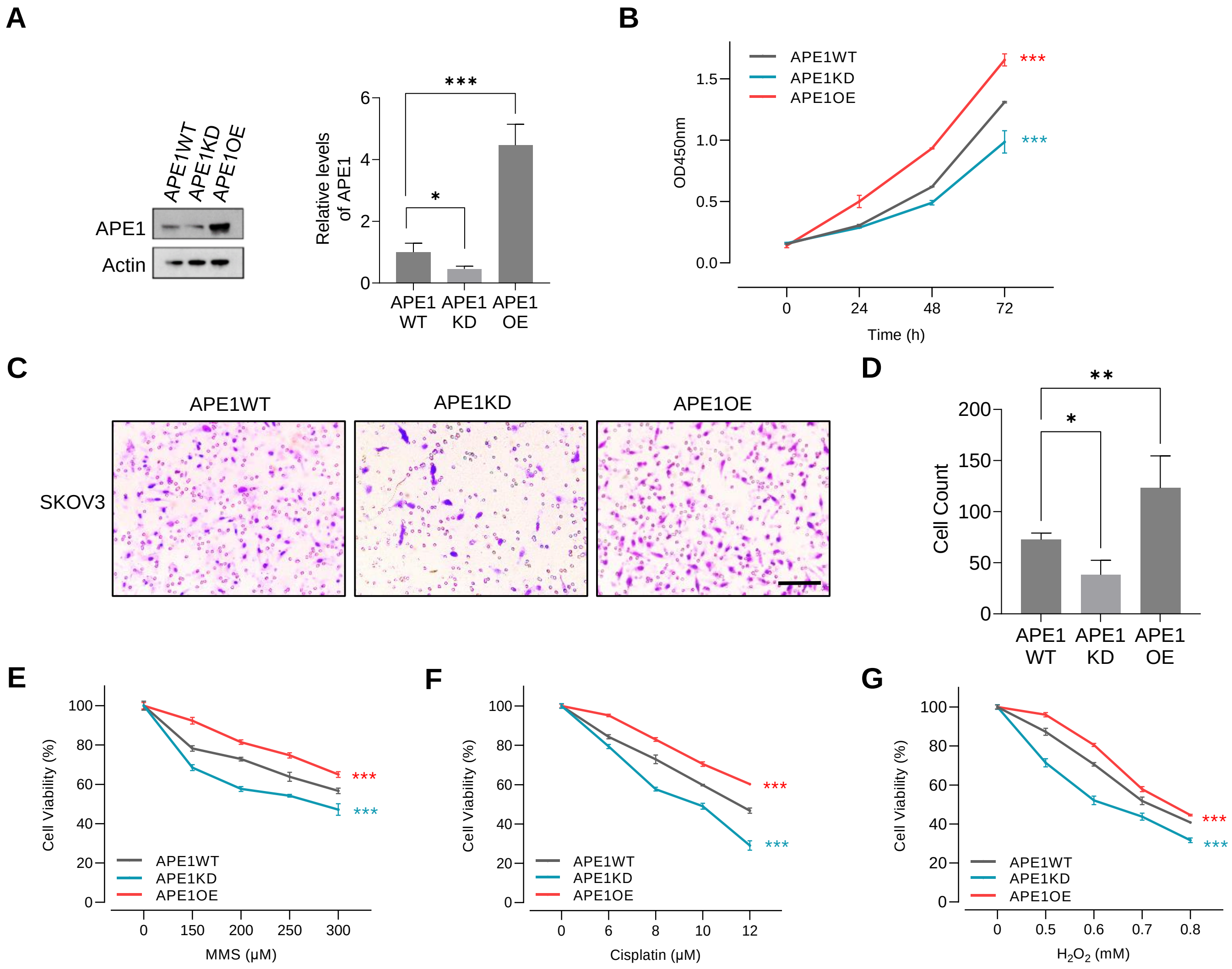

Supplementary Fig. 1 APE1 promotes cell proliferation and migration. **A** Representative western blot validation of APE1 protein abundance in APE1WT, APE1KD and APE1OE cells. One-way ANOVA was performed to analyze the relative abundance of APE1 in APE1KD and APE1OE cells, compared to APE1WT. Mean ± SEM is shown (n=3). **B** Proliferation curve of APE1WT, APE1KD and APE1OE cells. Two-way ANOVA was performed to evaluate the statistical significance. Mean ± SEM is shown (n=4). **C** The transwell assay was conducted to evaluate the migration ability of AEP1WT, APE1KD and APE1OE cells. Scale bar, 100 μm. **D** One-way ANOVA was performed on cell counts of the transwell assay. Mean ± SEM is shown (n=5). **E-G** The impact of APE1 on cell survival was determined by cell viability assays using the CCK-8 kit. APE1WT, APE1KD and APE1OE cells were treated with the indicated concentrations of MMS (E), cisplatin (F) and H_2O_2 (G) for 18 h, 24 h and 12 h, respectively. Two-way ANOVA was performed to evaluate the statistical significance. Mean ± SEM is shown (n=4)

Supplementary Fig. 2

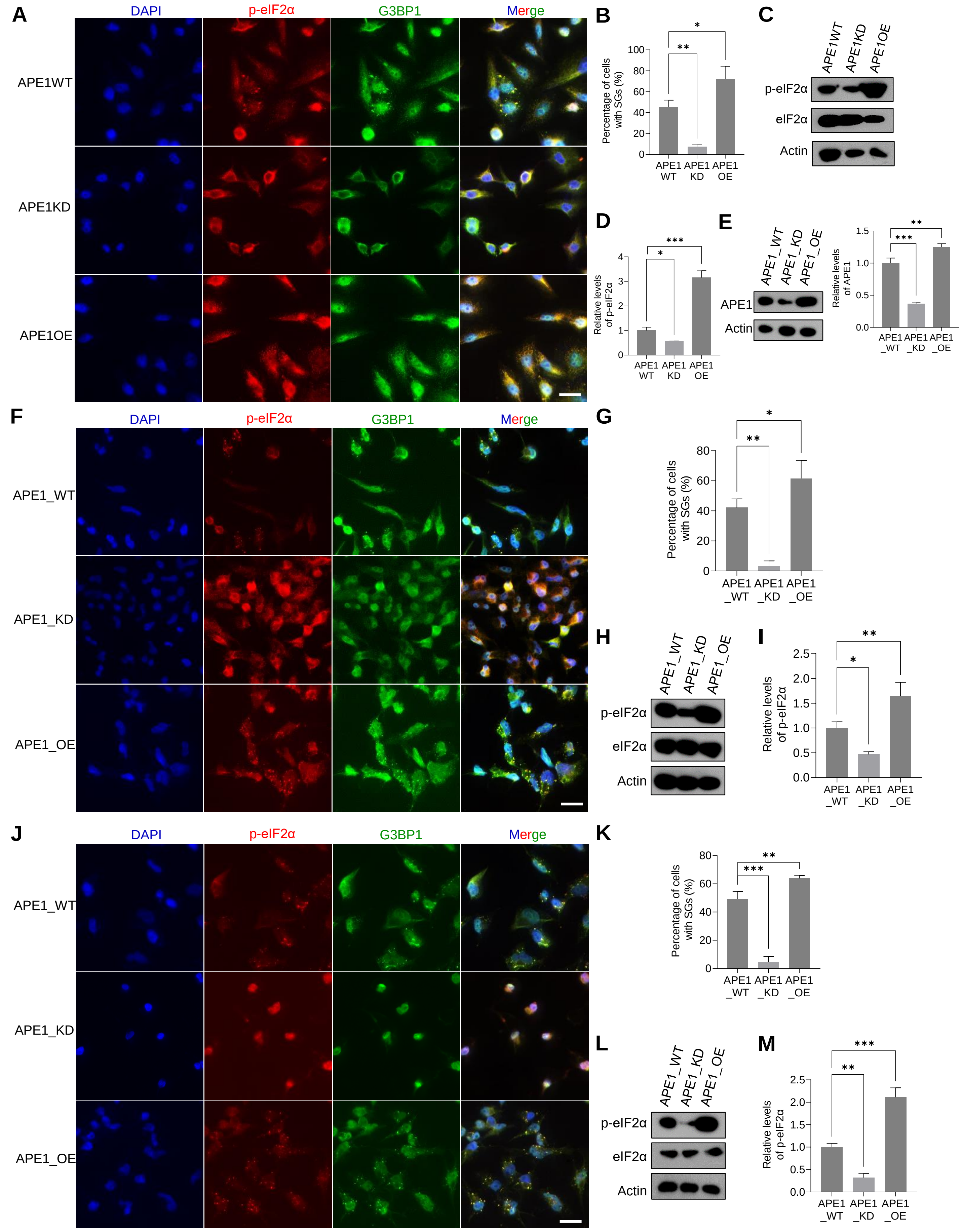

Supplementary Fig. 2 APEX1 promotes SG formation in cells. **A** Representative images of IF studies conducted in APE1WT, APE1KD and APE1OE cells after 250 μM cisplatin treatment for 4 hours. Coverslips were probed with p-eIF2α and G3BP1 antibodies to mark SGs. p-eIF2α (red) and G3BP1 (green) were merged with the nuclear stain DAPI (blue). Scale bar, 20 μm. **B** Quantification of the percentage of cells containing SGs in APE1KD and APE1OE relative to APE1WT. Data represent means ± SD of n = 3 independent replicates. **C** Representative western blot result shows the phosphorylation level of eIF2α in APE1WT, APE1KD and APE1OE cells after 250 μM cisplatin treatment. **D** Relative abundance of p-eIF2α in APE1KD and APE1OE cells compared to APE1WT is analyzed by One-way ANOVA. Mean ± SEM is shown (n = 3). **E** Representative western blot validation of APE1 protein abundance in APE1_WT, APE1_KD and APE1_OE cells. One-way ANOVA was performed to analyze the relative abundance of APE1 in APE1_KD and APE1_OE cells, compared to APE1_WT. Mean ± SEM is shown (n=3). **F** Representative images of IF studies conducted in APE1_WT, APE1_KD and APE1_OE cells after 0.5 mM H_2O_2 treatment for 3 hours. Coverslips were probed with p-eIF2α and G3BP1 antibodies to mark SGs. p-eIF2α (red) and G3BP1 (green) were merged with the nuclear stain DAPI (blue). Scale bar, 20 μm. **G** Quantification of the percentage of cells containing SGs in APE1_KD and APE1_OE relative to APE1_WT. Data represent means ± SD of n = 3 independent replicates. **H** Representative western blot result shows the phosphorylation level of eIF2α in APE1_WT, APE1_KD and APE1_OE cells after treatment. **I** Relative abundance of p-eIF2α in APE1_KD and APE1_OE cells compared to APE1_WT is analyzed by One-way ANOVA. Mean ± SEM is shown (n = 3). **J** Representative images of IF studies conducted in APE1_WT, APE1_KD and APE1_OE cells after 250 μM cisplatin treatment for 4 hours. Coverslips were probed with p-eIF2α and G3BP1 antibodies to mark SGs. p-eIF2α (red) and G3BP1 (green) were merged with the nuclear stain DAPI (blue). Scale bar, 20 μm. **K** Quantification of the percentage of cells containing SGs in APE1_KD and APE1_OE relative to APE1_WT. Data represent means ± SD of n = 3 independent replicates. **L** Representative western blot result shows the phosphorylation level of eIF2α in APE1_WT, APE1_KD and APE1_OE cells after 250 μM cisplatin treatment. **M** Relative abundance of p-eIF2α in APE1_KD and APE1_OE cells compared to APE1_WT is analyzed by One-way ANOVA. Mean ± SEM is shown (n = 3)

Supplementary Fig. 3

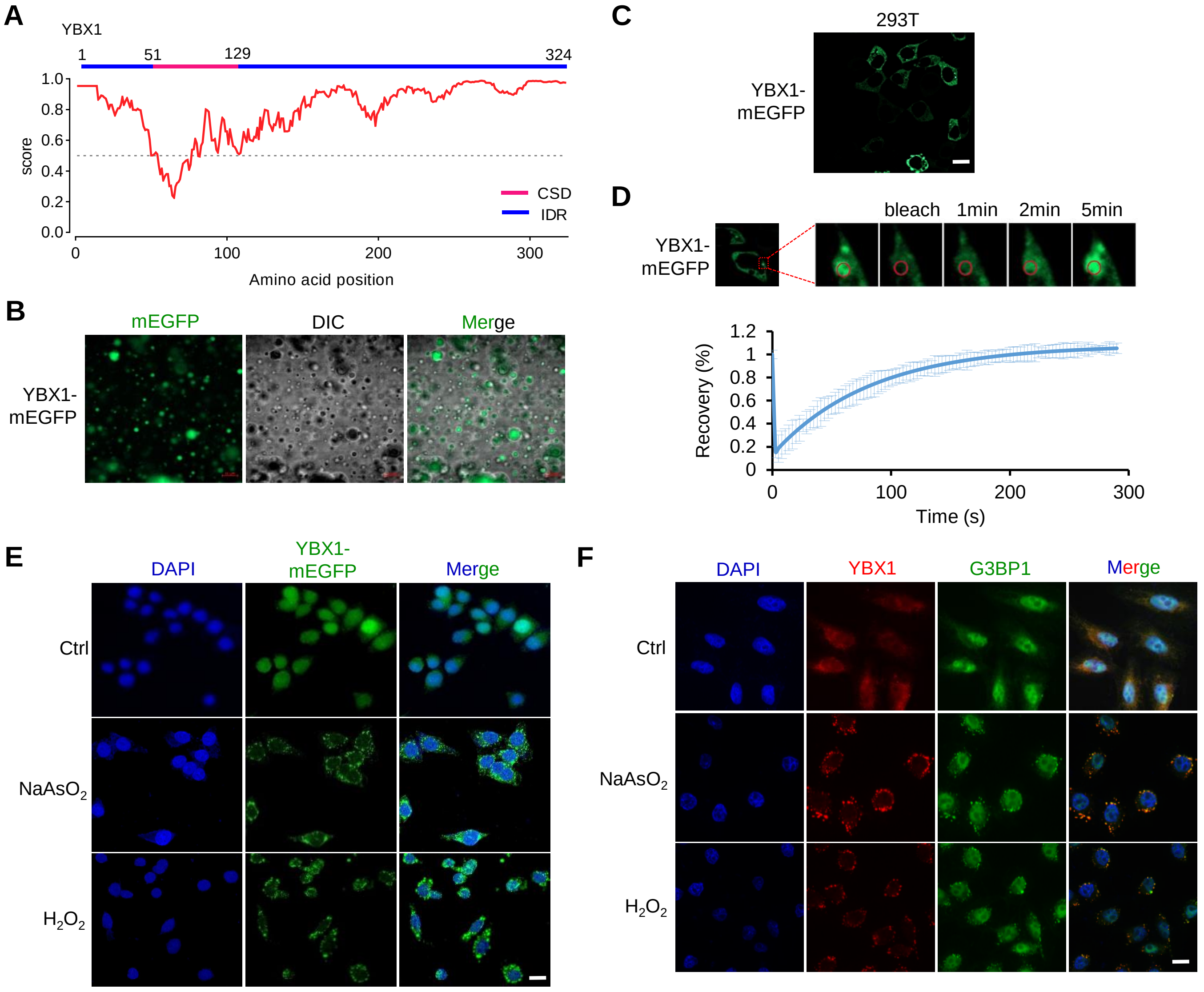

Supplementary Fig. 3 YBX1 forms biocondensates *in vitro* and *in vivo* and localizes to SGs. **A** YBX1 was predicted by IUPred3 to have an N-terminal intrinsically disordered region (IDR) and a C-terminal IDR, separated by a cold-shock domain (CSD). The score corresponds to the probability of the given residue being part of a disordered region. **B** *In vitro* phase separation assay was performed with purified monomeric enhanced GFP (mEGFP)-tagged YBX1. Phase separation of YBX1 was detected at concentration as low as 5 μM. Scale bar, 10 μm. **C** Live cell image of 293T cells transiently transfected with mEGFP-YBX1. Visualized under Zeiss LSM900 confocal microscope. Scale bar, 20 μm. **D** Fluorescence recovery after photobleaching (FRAP) experiments were performed on the cytoplasmic condensates of mEGFP-YBX1. Frap curve was created with Frapbot. **E** SKOV3 cells transfected with mEGFP-YBX1 were treated with 0.5 mM sodium arsenite or 0.5 mM H_2O_2 or left untreated and visualized under Zeiss LSM900 confocal microscope. Scale bar, 20 μm. **F** Representative immunofluorescence images show the colocalization of YBX1 (red) and G3BP1 (green) after treatment. Scale bar, 20 μm

Supplementary Fig. 4

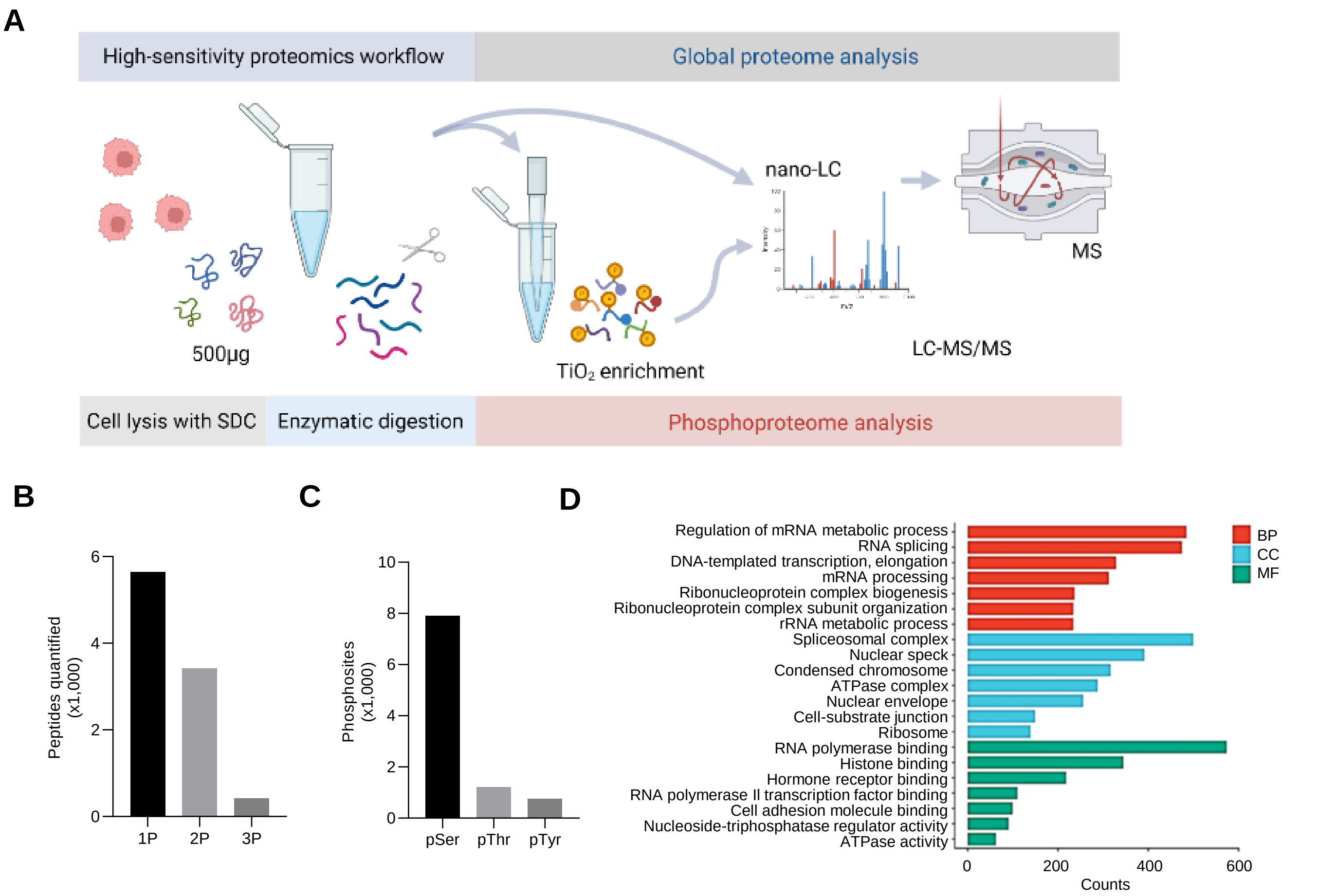

Supplementary Fig. 4 APE1 alters the phosphoprofile of SG proteins. **A** Workflow of high-sensitivity phosphoproteomic and global proteomic analysis performed on APE1WT, APE1KD and APE1OE cells. Label-free quantification was conducted on four replicates. **B** Quantification of the identified phosphopeptides with single, double and triple phosphorylation. **C** Quantification of the amino acid residues (serine, threonine, and tyrosine) modified by phosphorylation. **D** Gene ontology (GO) analysis was performed on all significantly regulated phosphoproteins ($p<0.01$, phosphopeptide abundance: APE1OE/APE1WT>2 and APE1KD/APE1WT<0.5; or $p<0.01$, APE1KD/APE1WT>2 and APE1OE/APE1WT<0.5). BP, biological process; CC, cellular component; MF, molecular function

Supplementary Fig. 5

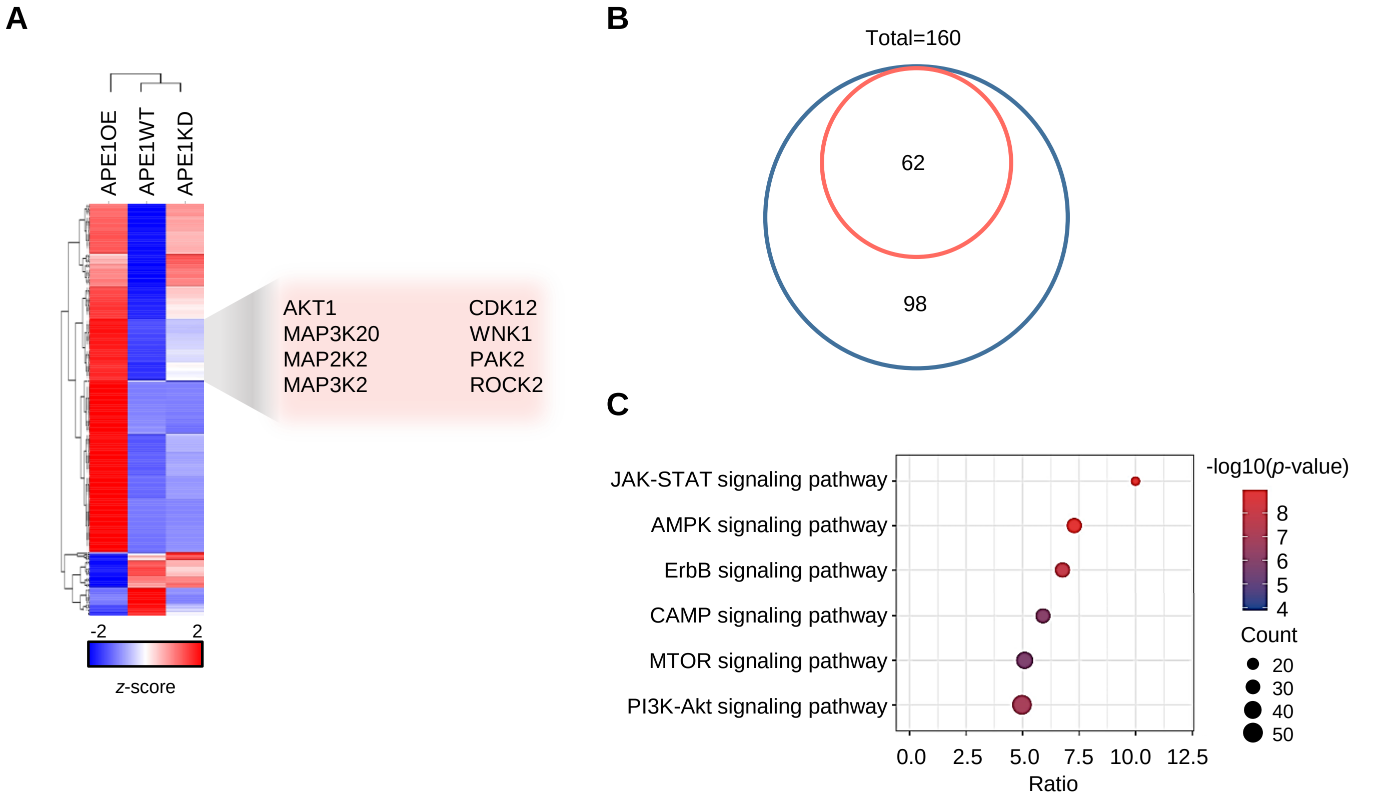

Supplementary Fig. 5 Phosphoprofile alteration of proteins involved in kinase signaling pathways. **A** Unsupervised hierarchical clustering of 276 significantly regulated phosphopeptides in APE1WT, APE1KD and APE1OE cells. Averaged replicates (n=4); heatmap shows *z*-scored $\log_2$-transformed phosphopeptide intensity. **B** A total of 160 proteins involved in kinase signaling pathways were significantly ($p<0.01$, phosphopeptide abundance: APE1OE/APE1KD>2 or <0.5) regulated by APE1, 62 of which are protein kinases. **C** Bubble plot representing enriched kinase signaling pathways

Supplementary Fig. 6

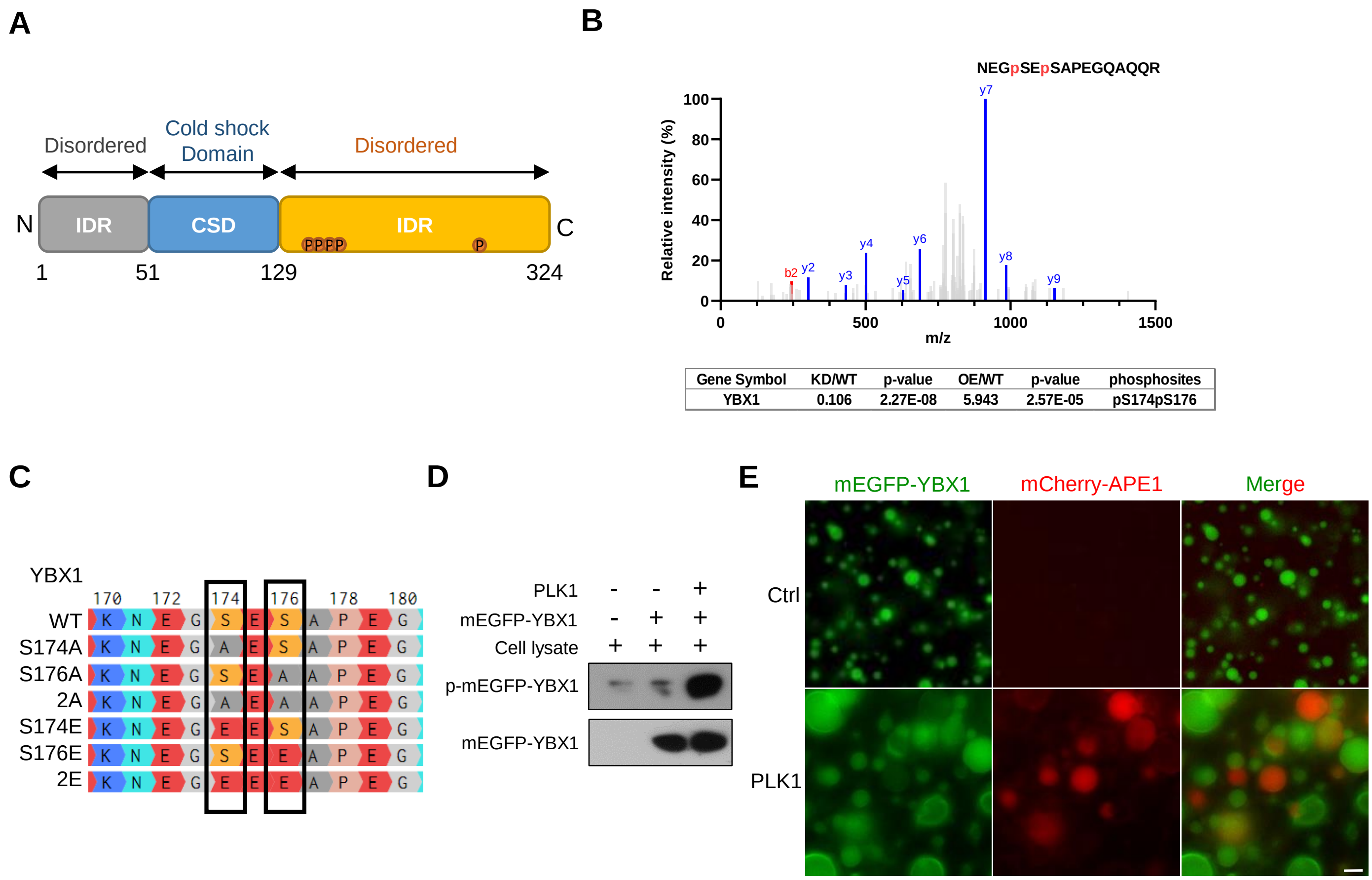

Gene Symbol	KD/WT	p-value	OE/WT	p-value	phosphosites
YBX1	0.106	2.27E-08	5.943	2.57E-05	pS174pS176

Supplementary Fig. 6 MS identification of phosphorylation of YBX1 at S174 and S176 residues. **A** YBX1 protein structure contains an N-terminal intrinsically disordered region (IDR), a cold-shock domain (CSD) and a C-terminal IDR. A phosphorylation cluster in the C-terminal IDR was denoted. **B** MS spectrum of the identified peptides of S174 and S176 double phosphorylation of YBX1. Fragment ion peaks are annotated in red and blue for b- and y-fragment ions, respectively. Table below shows the relative abundance of the phosphopeptide in APE1KD and APE1OE after quantification. **C** S174 and S176 were singly or doubly mutated to alanine (A) or glutamic acid (E). **D** *In vitro* phosphorylation assay was performed with purified mEGFP-YBX1 and PLK1. Immunoprecipitation of mEGFP-YBX1 was performed with anti-Flag M2 beads. Anti-phosphoserine antibody was used to assess the phosphorylation level of YBX1. **E** Phase separation assay was conducted using purified mCherry-APE1 and mEGFP-YBX1 from the phosphorylation assay.
